# Acceptance and Commitment Therapy for Children, Adolescents, and Young Adults With Cancer and Their Parents and for Children, Adolescents, and Young Adults Affected by Parental Cancer: A Population‐Stratified Systematic Review

**DOI:** 10.1002/brb3.71902

**Published:** 2026-11-09

**Authors:** Giorgio Alagna, Kim Prox, Charlotte Hanisch

**Affiliations:** ^1^ Psychology and Psychotherapy in Special Education & Rehabilitation, Faculty of Human Sciences University of Cologne Cologne Germany

**Keywords:** acceptance and commitment therapy, cancer, pediatric psycho‐oncology, systematic review

## Abstract

**Objective:**

Cancer profoundly affects the entire family system; evidence for acceptance and commitment therapy (ACT) remains fragmented within three distinct cohorts: (1) children, adolescents, and young adults (CAYAs) with cancer or cancer survivors, (2) CAYAs impacted by parental cancer, and (3) parents of CAYAs with cancer. This population‐stratified systematic review evaluated ACT's acceptability, feasibility, and efficacy.

**Methods:**

Adhering to Preferred Items for Systematic Reviews and Meta‐Analyses (PRISMA) 2020, 12 databases (including MEDLINE, PsycINFO, and Embase) and gray literature were searched from inception to October 2025. Quantitative studies evaluating ACT‐based interventions were included. Two independent reviewers screened studies, extracted data, and assessed methodological quality using JBI tools and the Risk of Bias in *N*‐of‐1 Trials (RoBiNT) Scale. Data were synthesized narratively by outcome within population strata following synthesis without meta‐analysis (SWiM), with effectiveness appraised for certainty of evidence using GRADE (Grading of Recommendations Assessment, Development, and Evaluation).

**Results:**

Nine studies (*N* = 573) were included (one RCT, one single case experimental design [SCED], four quasi‐experimental trials, and three single‐group pilot/feasibility studies). No studies targeted children. For AYA populations (both with cancer or cancer survivors and affected by parental cancer), evidence was of very low certainty; psychological flexibility emerged as a plausible mediator of quality‐of‐life improvements in a single mixed sample study of distressed AYAs affected by their own or a family member's cancer. Moderate‐certainty evidence for parents of CAYAs with cancer showed reductions in distress, anxiety, and depressive symptoms (*d* = 0.61–0.83), and increased psychological flexibility (*d* = 0.63).

**Conclusions:**

ACT shows promise for improving mental health outcomes in parents of CAYAs with cancer. Evidence for both AYAs populations remains limited by methodological heterogeneity, highlighting a need for developmentally tailored, rigorous trials.

## Graphical abstract

This systematic review included nine studies (N = 573) of acceptance and commitment therapy (ACT) for families affected by childhood, adolescent and young adult cancer. For parents of children and young people with cancer, ACT reduced distress, anxiety and depression (d = 0.610.83) and improved psychological flexibility, with moderate certainty. For adolescents and young adults with cancer or affected by parental cancer, the evidence was of very low certainty. ACT shows promise for parents, while developmentally tailored trials for young people are needed.

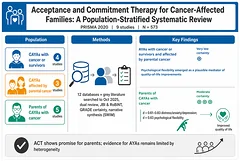

## 1 Introduction

### 1.1 The Psychosocial Burden of Cancer in Youth and Families

The World Health Organization estimates that each year worldwide, “400,000 children and adolescents of 0–19 years old develop cancer” (World Health Organization: WHO 2025). Although many childhood cancers are now effectively treated—with survival rates approaching 80% in high‐income countries (World Health Organization: WHO 2025)—cancer remains a significant predisposing factor for long‐term psychological distress and mental disorders (Hsu et al. 2023). These consequences on mental health stem from multiple sources such as long‐term side effects of cancer treatment (Eiser 1998), disruption of age‐appropriate developmental tasks (Schröder et al. 2019), and chronic stressors such as fear of cancer recurrence (FCR) (Yang et al. 2019), intrusive memories (Tremolada et al. 2016), social isolation, and elevated school absenteeism (French et al. 2013).

However, such biopsychosocial consequences do not occur in a vacuum; they unfold within the child's primary social environment. Consequently, psychosocial interventions in pediatric psycho‐oncology comprise not only young cancer patients, but also the entire family system affected by the diagnosis (Schröder et al. 2019; Neugebauer and Mastergeorge 2021). For primary caregivers of a child with cancer, the diagnosis and treatment can lead to elevated stress, clinically significant anxiety, depression, and posttraumatic stress (Sultan et al. 2016), impaired quality of life (QoL) (Klassen et al. 2011), and financial burdens due to lost work time, unexpected hospital stays, and homecare needs (Neugebauer and Mastergeorge 2021). In the inverse situation, when children, adolescents, and young adults (CAYAs) are affected by parental cancer, they face elevated psychological distress 3–6 times higher than age‐matched peers (Patterson et al. 2017), including internalizing and externalizing problems, stress, and social isolation, with parentification and disruption of developmental tasks as key mechanisms (Visser et al. 2004; Morris et al. 2016; Walczak et al. 2018; Wray et al. 2022).

### 1.2 Acceptance and Commitment Therapy (ACT) as a Transdiagnostic Framework

With cancer's multifaceted consequences across the family system, clinicians face heterogeneous stressors, intervention targets, delivery constraints, outcome priorities, and plausible mechanisms of change. Against this background, a transdiagnostic framework capable of addressing these diverse presentations through unifying change mechanisms might be particularly valuable. ACT is such a framework: As intervention grounded in contextual behavioral science, ACT offers a process‐based model centered on psychological flexibility—defined as the capacity to remain in contact with the present moment and to act values‐based even in the presence of distress (Feros et al. 2013)—which represents a unified mechanism of change across diverse presentations (Hayes et al. 2006; Hayes 2004).

ACT's theoretical model distinguishes primary suffering or “clean” pain (the unavoidable distress, e.g., distress inherent to a serious illness) and secondary suffering or “dirty” pain, which arises from dysfunctional attempts to control or avoid internal private experiences (e.g., rumination, experiential avoidance) (Stoddard and Afari 2014). ACT's mission is to address the “dirty” pain through six interrelated processes—acceptance, cognitive defusion, mindfulness, self‐as‐context, clarifying values, and committed actions (Hayes et al. 2006)—emphasizing not the elimination of difficult thoughts and emotions, but a change in how individuals relate to them, enabling continued value‐aligned action despite unavoidable distress.

### 1.3 ACT and Psycho‐Oncology

ACT may be particularly suited to populations confronting life‐threatening illness, as it addresses existential distress, unavoidable suffering, and value‐based engagement (Alder 2020). Cancer patients are exposed to particular negative internal experiences shaped by a pervasive sense of loss of control (Boer et al. 1998), which acceptance (Gong et al. 2019), present moment focus, and clarity about personal values (Zhao et al. 2021) are well suited to address. Crucially for the CAYAs impacted by cancer, ACT's process‐based model maps directly onto key psychosocial challenges: Acceptance and defusion may address FCR, intrusive memories, serving as emotion regulation strategies (López et al. 2021; Maulana and Rangkuti 2025); self‐as‐context supports identity reconstruction beyond the cancer experience (Alder 2020); and values‐based committed action may reduce school avoidance and increase class attendance (Takahashi et al. 2020; Petersen et al. 2023).

In adult psycho‐oncological populations, meta‐analytic evidence shows that ACT reduces depression, anxiety, FCR, and psychological distress, while improving quality‐of‐life and psychological flexibility (Sauer et al. 2024). Psychological flexibility has been shown to mediate improvements in emotional difficulties and QoL directly, supporting its role as an active mechanism of change (Sauer et al. 2024; González‐Fernández and Fernández‐Rodríguez 2019).

### 1.4 Current Evidence Gaps and Developmental Considerations

Despite these promising findings, the evidence base for ACT in psycho‐oncology carries important limitations. The umbrella review by Sauer et al. (2024) found that the included reviews and meta‐analyses were of low or critically low methodological quality. Effects were inconsistent for physical outcomes such as fatigue and sleep disturbance, and appeared attenuated in advanced‐stage disease, suggesting that ACT's benefits may not generalize uniformly across the cancer trajectory. Furthermore, few studies have directly compared ACT against other established psychosocial interventions (e.g., CBT, mindfulness‐based stress reduction) in cancer populations, leaving its incremental value relative to existing approaches largely unestablished.

Moreover, the applicability of the depicted adult findings to CAYAs is uncertain. CAYAs differ from adult patients in developmentally salient ways—including caregivers dependence, centrality of peer relationships, vulnerability regarding normative milestone attainment, and generalization of strategies’ functionality across contexts (Schröder et al. 2019; Petersen et al. 2024; Wicksell et al. 2015). Therefore, relevant mediators such as psychological flexibility, acceptance, and values‐aligned behavior identified in adult psycho‐oncology (Sauer et al. 2024) might operate differently across these populations, requiring tailored delivery adaptations (Petersen et al. 2024; Wicksell et al. 2015). ACT with children and adolescents must account for these differences through required adaptations organized along biopsychosocial developmental considerations (Halliburton and Cooper 2015). Biologically, ongoing neurocognitive maturation manifests as shorter sustained attention and greater motor restlessness, so that interventions use behavioral activation and playful and experiential exercises rather than verbal discussion alone (Halliburton and Cooper 2015). Psychologically, the repertoires ACT recruits are still consolidating: Metaphor comprehension increases stepwise across middle childhood (Willinger et al. 2019), effortful emotion regulation strategies such as acceptance and cognitive reappraisal are constrained by maturing cognitive functioning (Willner et al. 2022; Troy et al. 2018) and may be substituted by greater reliance on avoidance‐based and externally scaffolded regulation, and the deictic relational repertoire underpinning perspective taking is age‐graded into adulthood (McHugh et al. 2004). Socially, it requires accounting for the salience of peer influence and identity formation during this developmental period, alongside family‐related factors: Self‐as‐context work engages a self‐concept still under construction, peers function as both therapeutic resource and competing influence, and adult‐initiated referral means intrinsic motivation must be built within therapy while adherence depends on caregiver‐supported attendance (Halliburton and Cooper 2015; Waumans et al. 2023; Coyne et al. 2011; Whittingham and Coyne 2019).

Practically, these considerations translate into required flexibility in delivering ACT's core processes (Halliburton and Cooper 2015). Values clarification should account for autonomy‐seeking behavior and peer pressure. Mindfulness exercises should be adapted to shorter attention spans, using activities such as mindful walking or eating. Self‐as‐context exercises should account for the critical period of identity formation, with acceptance potentially used to increase tolerance of identity‐related distress. Committed action may need to be coordinated with parents or caregivers. Defusion should rely on interactive, playful exercises rather than direct verbal challenge, as more confrontational approaches risk being perceived by youth as dismissive of their concerns (Turrell and Bell 2016).

Furthermore, working with CAYAs affected by cancer necessitates engaging their caregivers, since parents shape the contingencies through which children's coping behaviors are reinforced or extinguished (Petersen et al. 2024; Coyne et al. 2011). Parental psychological well‐being is a known contributor to children's adjustment to a cancer diagnosis (Bakula et al. 2019), and high parental experiential avoidance is associated with greater emotional and behavioral problems in children, constituting a risk factor for poorer child outcomes (Whittingham and Coyne 2019; Cernvall et al. 2016), making parental psychological flexibility a treatment target in its own right (Kearney et al. 2015). This interdependence is also reflected in how ACT‐based interventions for children and adolescents are typically delivered: Treatment is frequently provided by the same clinician, within the same treatment period, and at times within the same joint session involving both child and parent (Whittingham and Coyne 2019; Turrell and Bell 2016; Black 2022; Bibby et al. 2024).

### 1.5 Objectives of the Current Review

Considering the theoretical suitability of ACT for CAYAs affected by cancer or by parental cancer and the necessity to engage caregivers in psychological treatment of CAYAs affected by cancer, the objective of this review is to evaluate the acceptability, feasibility, and efficacy of ACT‐based interventions in three populations: (1) CAYAs (< 25 years) navigating their own cancer diagnosis or survivorship; (2) CAYAs impacted by parental cancer; and (3) parents or primary caregivers of CAYAs affected by cancer. Although these subpopulations differ meaningfully with respect to stressor types and intervention contexts, synthesizing the evidence within a population‐stratified framework advances a holistic approach to psycho‐oncologic care in/with/within families. This strategy not only identifies differential evidence quality and priority gaps for future trials, but also yields actionable, person‐centered interventions to address the psychological responses of the entire family system.

## 2 Methods

This systematic review was registered on the PROSPERO registration platform (CRD420251104115) and follows the PRISMA (Preferred Items for Systematic Reviews and Meta‐Analyses) reporting guidelines (Page et al. 2021).

### 2.1 Eligibility Criteria

The PICOS criteria for this review were

Population:

‐ CAYAs (aged < 25 years) who are either
‐ Diagnosed with cancer and currently undergoing treatment, or
‐ Pediatric cancer survivors (any time posttreatment);
‐ CAYAs (aged < 25 years), whose parents or primary caregivers have cancer and are currently undergoing treatment;
‐ Parents or primary caregivers of CAYAs (aged < 25 years) with cancer or who are childhood cancer survivors.

Intervention: Studies were eligible if ACT or its adaptations were the primary intervention.

Comparator: No restrictions were placed on the type of control or comparison group; studies with active controls, passive controls, or no control group were all eligible.

Outcomes: Eligible studies were required to report at least one psychosocial or health‐related outcome assessed using a psychometric instrument. We did not restrict inclusion based on the specific instrument used.

Study design: Study designs with quantitative outcomes were eligible, including (but not limited to) randomized controlled trials, nonrandomized trials, pre‐post studies, and single‐case experimental designs.

No restrictions were placed on language, publication year, follow‐up duration, or setting. Non‐English/Italian/German texts were translated via DeepL (DeepL Translator n.d.). Gray literature was eligible.

This broad approach to study design was adopted given that ACT‐based interventions for the three populations represent an emerging area of research, where rigorous randomized controlled trials remain scarce. Restricting eligibility to controlled designs alone would have excluded the majority of the available evidence, particularly for AYA‐focused strata where research is still at the feasibility and pilot stage.

This approach, however, introduces substantial heterogeneity in methodological rigor. We addressed this by retaining study design and risk of bias as explicit qualifiers throughout the narrative synthesis (Section 3.4), and by formally incorporating them into GRADE (Grading of Recommendations Assessment, Development, and Evaluation) certainty‐of‐evidence ratings (Section 2.8), allowing readers to appropriately weigh findings by methodological strength.

### 2.2 Searching Strategy

A comprehensive search was conducted in MEDLINE, CINAHL, EMBASE, APA PsycInfo, PsyIndex, LIVIVO, Web of Science, Europe PMC, WHO International Clinical Trials Registry Platform, PubMed, and clinicaltrials.gov. Gray literature was searched in DANS Data Station (Life Sciences; Social Sciences and Humanities) and EThOS; ResearchGate was also consulted. The selection of the databases was integrated through the recommendations of Search Smart AI. The databases and registries were last searched between October 11, 2025 and October 22, 2025. Keywords and medical subject headings were chosen following the PICO criteria (e.g., “child*,” “adolescent*,” “parent*,” “Acceptance and Commitment Therapy,” “cancer,” “neoplas*”). The keywords were written in English, German, and Italian, respecting the language knowledge of the team. Full search strings for each database and registry can be found in the OSF Project at: https://osf.io/qd6×7. The reference list of included studies was screened for potentially eligible studies. Citation tracking was conducted via ResearchRabbit AI (ResearchRabbit AI n.d.) and Scite AI (Scite Browser Extension n.d.); related PROSPERO reviews were also screened. Three experts (two authors of included studies and an author of a study protocol) were contacted to identify additional eligible studies.

### 2.3 Selection Process

All retrieved studies were uploaded into EndNote 21 (The EndNote Team 2013) and deduplicated automatically, followed by manual verification. Two independent reviewers (G. A. and K. P.) screened each study independently at every stage. In case of disagreement, the two reviewers consulted a third reviewer (C. H.) to reach a consensus.

### 2.4 Data Collection

A standardized extraction table was created and tested with three studies. Data were extracted independently by two reviewers (G. A. and K. P.) and cross‐checked using Claude Sonnet 4.5 via Perplexity Pro (Perplexity n.d.), which provided the study protocol, PROSPERO entry, PRISMA 2020 checklist, and *Cochrane Handbook* Chapter 5 (Cumpston et al. 2019) as context. To minimize hallucination risk, human extraction was completed prior to consulting the AI output (human‐in‐the‐loop approach). Data from multiple reports linked to one study (e.g., study protocol, feasibility, and acceptability studies) were collapsed, indicating in the extraction table the most complete study. Diverging data from multiple reports were reported separately for each collapsed report. We did not contact the authors for missing data.

### 2.5 Data Items

All outcomes reported in the studies were extracted. Means and standard deviations were reported for the two extreme timepoints (e.g., T0 and T1 for studies with only one follow‐up, T0 and T2 for studies with two follow‐ups). Data extraction prioritized the primary endpoint appropriate to the study's developmental phase. For definitive trials aimed to detect clinical change, we prioritized the extraction of efficacy outcomes (psychosocial and health‐related). For pilot and feasibility studies where efficacy was not the primary aim, we extracted feasibility and acceptability data to characterize the preliminary evidence base. Participants’ characteristics (total number, age, diagnosis, and time since cancer diagnosis), study design, timepoints of data collection, session adherence, *N* at each timepoint, and companion reports were also reported. Funding sources of each study can be found in the OSF project. When data were missing or unclear and could not be computed from available information, it was reported as “not reported.”

### 2.6 Risk of Bias Assessment

Risk of bias was assessed in all studies, except process evaluation studies (e.g., evaluating only acceptability and feasibility), using the JBI critical appraisal tool for quasi‐experimental studies (Barker et al. 2024), the JBI critical appraisal tool for randomized controlled trials (Barker et al. 2023), and the Risk of Bias in *N*‐of‐1 Trials (RoBiNT) Scale (Tate et al. 2013).

Since the JBI tools do not provide a standardized algorithm for deriving overall risk of bias judgments, studies rated with these tools were classified as high, moderate, or low risk of bias based on a priori criteria applied to critical domains (measurement of outcomes, selection and allocation, confounding, participant retention).

Studies were rated high risk of bias if they had any “No” response in a critical domain, two or more “No” responses across other major‐concern domains, or serious methodological flaws that fundamentally compromised internal validity. Studies were rated moderate risk of bias if they had “Yes” responses to all critical domains but three or more “No/Unclear” responses across other domains, or methodological limitations that could affect the findings without invalidating potential inferences. Studies were rated low risk of bias if they had “Yes” responses to all critical domains, with no more than three “No/Unclear” responses in noncritical domains (e.g., blinding, which is often infeasible in psychological interventions).

The risk of bias was assessed independently by two reviewers (G. A. and K. P.). In case of disagreement, the two reviewers consulted a third reviewer (C. H.) to reach a consensus.

### 2.7 Effect Measures and Synthesis Methods

Standardized mean differences (Cohen's *d*) with 95% confidence intervals were calculated for continuous outcomes. Between‐group effect sizes were calculated for controlled trials, and within‐group effect sizes were calculated for single‐group designs. When *η^2^* was reported by the study's authors, this outcome measure was stated in the table after the calculation of Cohen's *d* between groups. For moderation and mediation analyses, means and standard deviations at each timepoint were extracted where possible to compute Cohen's *d*. Effect sizes were interpreted using conventional benchmarks (small: 0.2; medium: 0.5; large: 0.8). Meta‐analysis was not planned a priori due to anticipated heterogeneity in populations, interventions, and study designs.

This anticipated heterogeneity was evaluated qualitatively, given that formal heterogeneity testing presupposes a pooled quantitative synthesis, which was not appropriate here due to the conceptual and methodological diversity across the three population strata (Section 2.1). Studies were synthesized narratively, structured by population group and outcome domain. Effectiveness was evaluated through standardized clinical outcomes and appraised via GRADE. Acceptability and feasibility outcomes (e.g., satisfaction, implementation fidelity) were summarized narratively when reported as the primary outputs of pilot or feasibility studies, while session attendance was reported descriptively across all studies where available. Narrative synthesis followed the synthesis without meta‐analysis (SWiM), as specified in the a priori protocol. Where sufficient data were available to draw meaningful conclusions, key findings were summarized in summary of findings (SoF) tables, detailing the range of observed effects and the associated GRADE certainty of evidence ratings for critical outcomes.

### 2.8 Reporting Bias and Certainty Assessment

Risk of bias due to missing results was planned using funnel plots or statistical tests.

Certainty in the body of evidence was assessed by one reviewer (G. A.) using the GRADE assessment, guided by a standardized checklist (Meader et al. 2014) to ensure consistency and reproducibility. Assessments were conducted separately for each population group to account for clinical heterogeneity. A second reviewer (K. P.) audited the assessments a posteriori, and any disagreements were resolved through consultation with a third reviewer (C. H.). The GRADE‐outcomes were selected out of the critical outcomes assessed through psychometric instruments depicted in the study protocol and can be found in the OSF Project.

Starting certainty ratings were determined by the level of the dominant study design (HIGH for RCTs, LOW for quasi‐experimental designs, and VERY LOW for pre‐post studies with no control group). Certainty was downgraded for risk of bias, inconsistency, indirectness, imprecision, or publication bias (by one or two levels per domain), and upgraded for large effect sizes (*d* > 0.8), dose–response gradients, or plausible residual confounding reducing the observed effect.

GRADE certainty ratings ranged from very low to high, reflecting confidence that the true effect is close to the estimated effect.

## 3 Results

### 3.1 Study Selection

A total of 367 records were identified through searching in databases and registers. After deduplication, the title and abstract of 215 studies were screened, and the full text of 54 studies was screened for eligibility, since the full text of 7 studies couldn't be accessed. Twenty studies were excluded because they didn't satisfy the population criteria, since the population was affected not only by cancer (Burke et al. 2014; Van Hall 2014; Rayner et al. 2016). Eight studies were excluded because the core intervention wasn't ACT (Smith et al. (2015) used CBT and RFT) or used ACT mixed with other interventions, entering confounding factors (Kaiser (2012) used a combination of ACT and MBCT). Six studies were excluded because they were only protocols without results. The authors of protocols that may have been eligible were contacted to ask if results were ready (Malins et al. 2021), so as to include the studies, but no result was ready. Through other methods, 49 reports were found, of which 11 were considered suitable, but were already found through database and registry searching. The final inclusion consisted of 17 reports representing 9 studies, of which 8 were companion reports (Table S1). Inter‐rater reliability for full‐text screening was high (agreement = 94.4%, *k* = 0.88) (Figure 1). Notable near‐eligible studies included Martin et al. (2021), excluded because their population had neurofibromatosis Type 1 rather than cancer per se, and Malins et al. (2021), an ongoing RCT for brain tumor survivors whose results were unavailable. Three studies targeted AYAs with cancer or survivors (Stuart 2022; Patterson et al. 2022; Clarke et al. 2021), one study targeted AYAs impacted by parental cancer (Bibby et al. 2024), and five targeted parents (Baratian et al. 2017; Akbari et al. 2019; Bautista et al. 2023; Kalateh et al. 2024; Jin et al. 2025).

**FIGURE 1:**
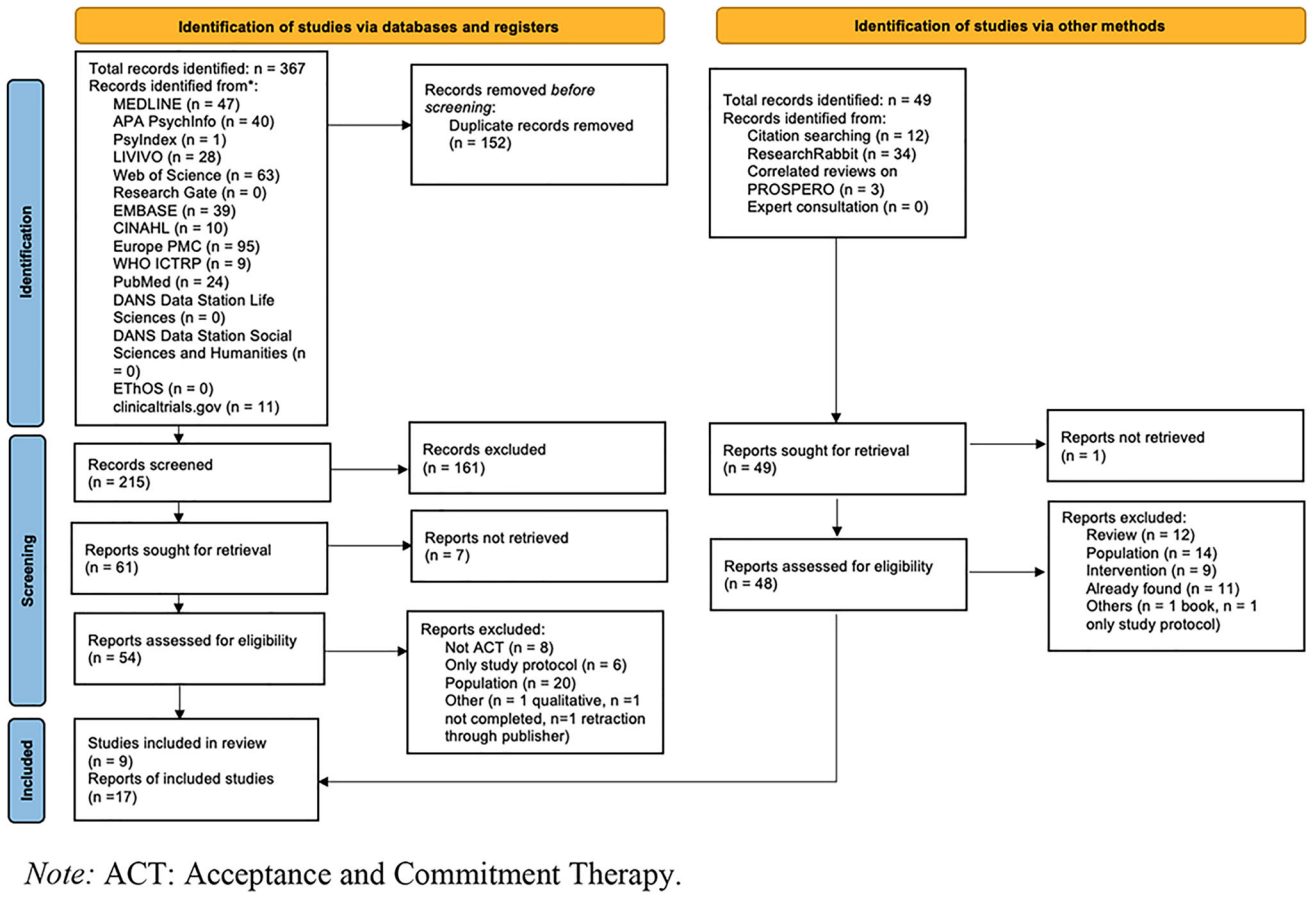
PRISMA 2020 flow diagram for study retrieval and selection. Abbreviation: ACT, acceptance and commitment therapy.

### 3.2 Study Characteristics

Table 1 encompasses the name of the authors, characteristics of participants, characteristics of intervention, timepoints of data collection, measurement tools, outcomes, results, session attendance, and loss to follow‐up of the nine studies included in the review. Nonsignificant outcomes and companion reports are not reported in the extraction table but can be found in the OSF Project. Seven of the studies were in English, two in Farsi (Baratian et al. 2017; Akbari et al. 2019). All studies were published in the last 10 years.

**TABLE 1:** Extraction table of included studies.

Author, year, and country/region	Study design	Participants characteristics (*N*, mean age, SD and range, diagnosis condition, time since diagnosis)	Description of the intervention (session number, duration of a session, frequency, delivery mode, provider qualifications, control group)	Timepoints of data collection (except screening)	Quantitative outcomes and measurements tools (except screening)	Results (only significant: *p* < 0.05)	(Session) attendence, *N* at each timepoint
Clarke et al. (2021) **Australia**	Single group process evaluation	***N*** = 28 Adolescents who had been diagnosed with cancer, and who had completed curative cancer treatment or were receiving long‐term or maintenance therapy **Age**: *M* (SD) = 15.4 (1.6) **Condition**: Cancer (96%), 4% similar treatment (chemotherapy) without cancer diagnosis **Time since diagnosis**: Any (53% adolescence)	**Dose**: 90 min, five sessions over 3 days. **Delivery mode**: Camp‐based intervention. **Provider qualification**: Psychosocial workers with general ACT training.	End of each session	(1) Program acceptability (author‐made satisfaction questionnaires). (2) Program feasibility (author‐made implementation fidelity questionnaires only for the facilitators).	(1) Only process evaluation. Acceptability: 85% rated sessions as helpful, 89% as meaningful. (2) Implementation fidelity: 70% activities delivered as per manual; 39% of facilitators self‐reporting very/extremely competent delivery.	Attendence: 97%. *N* = 28 at each timepoint.
Patterson et al. (2022) **Australia**	Pre‐post study with no control group	***N*** = 148 (full evaluation cohort; analytic subsample *n* = 52). Distressed adolescents impacted by their own or parental cancer **Age**: *M* (SD) = 14.5 (1.6) **Condition**: Cancer or family member's cancer **Time since diagnosis**: Any type or stage of cancer in the previous 5 years	**Dose**: 90 min, 4 days. **Delivery mode**: Group, camp‐based. **Provider qualification**: Psychosocial workers with ACT training.	T0: Baseline T1: Immediately post‐program (QoL only) T2: 2 months after T1	(1) QoL (author‐made questionnaire). (2) Psychological distress (K10). (3) Peer support (CaPSS). (4) Psychological inflexibility (AFQ‐Y8). (5) Self‐compassion (SCS). (6) Mindfulness (CAMM).	(1) Quality of life: 20.3% experienced clinically meaningful improvement T0 → T1 (*d* ≥ 0.5). 15.0% experienced clinically meaningful improvement T0 → T2 (*d* ≥ 0.5) (2) Psychological distress: 18.0% experienced clinically meaningful improvement T0 → T2 (*d* ≥ 0.5). No significant mean‐level change reported. *M*/SDs not reported; effect sizes cannot be calculated. (3) Psychological distress significantly mediated QoL improvement (*β* = 0.77, SE = 0.20, 95% CI [0.39, 1.19]).	Attendence: not stated. Assessed for eligibility: *N* = 158. Inclusion criteria met: *N* = 148. T0: *N* = 148; T1: *N* = 148; T2: *N* = 102.
						(4) Psychological inflexibility: Data collected but detailed results not reported. Psychological flexibility significantly mediated QoL improvement (*β* = 0.21, SE = 0.11, 95% CI [0.002, 0.442]). *M*/SDs: Not reported; effect sizes cannot be calculated. (5) Self‐compassion: data collected but detailed results not reported. Self‐compassion (self‐kindness) significantly mediated QOL improvement (*β* = 0.26, SE = 0.12, 95% CI [0.04, 0.51]). *M*/SDs not reported; effect sizes cannot be calculated.^a^	
Stuart (2022) **USA**	Pre‐post study with no control group	***N*** = 11 adolescents and young adults who survived childhood cancer^b^ **Age**: *M* (SD) = 21.4 (2.17) **Condition**: Cancer **Time since diagnosis**: Any	**Dose**: 90 min, weekly. **Delivery mode**: Group, online. **Provider qualification**: Not reported.	T0: Before the intervention T1: After the fifth session	(1) Depression (PHQ‐9), (2) generalized anxiety (GAD‐7), (3) posttraumatic stress (PCL‐5), (4) interpersonal skills (IF), (5) neurocognitive functioning (NSSR), (6) cognitive flexibility (CFS), (7) health‐promoting behaviors (HB).	No significant outcome.	Attendence: 100%. Assessed for eligibility: *N* = 445. Inclusion criteria met: *N* = 205. T0: *N* = 11. T1: *N* = 9.
Bibby et al. (2024) **Australia**	Pragmatic controlled trial (nonrandom allocation)	***N*** = 142 AYAs with a parent who was receiving treatment for cancer or had been diagnosed with cancer (EG: *N* = 77, CG(WL): *N* = 65) **Age**: EG: *M* (SD) = 16.84 (2.82), CG: *M* (SD) = 17.11 (2.45) **Condition**: Parental cancer **Time since diagnosis**: In the last 5 years	**Dose**: 120 min, weekly. **Delivery mode**: Group, in‐person (17 groups) or online (4 groups). **Provider qualification**: Psychosocial workers with ACT training.	T0: Baseline T1: After intervention T2: 2 months after T1	(1) Unmet needs (OCNI). (2) Psychological distress (K10). (3) Psychological inflexibility (AFQ‐Y8). (4) Mindfulness (CAMM). (5) Family functioning (FRI).	(1) Unmet needs ↓ (intervention: T0 *M* (SD) = 103.66 (30.70), T2 *M* (SD): not reported (estimated *M* = 87.9). Control: T0 *M* (SD) = 100.85 (35.11), T2 *M* (SD): not reported (estimated *M* = 94.6); between‐group: *d* = 0.29 [−0.04, 0.62])^c^.	Attendence: 82.9%. Assessed for eligibility: *N* = 158. Inclusion criteria met: *N* = 147. EG: T0: *N* = 77; T1: *N* = 55; T2: *N* = 48. CG: T0: *N* = 65; T1: *N* = 47; T2: *N* = 37.^d^
**Akbari et al. (2019)** **Iran**	Quasi‐experimental with control group	***N*** = 30 mothers of children with cancer (EG: *N* = 15, CG(WL): *N* = 15) **Age**: EG: *M* (SD) = 37.27 (2.86), CG: *M* (SD) = 36.60 (2.87) **Condition**: Child's cancer **Time of diagnosis**: Any	**Dose**: 120 min, weekly. **Delivery mode**: Group, in‐person. **Provider qualification**: Clinical psychologists trained in ACT.	T0: Baseline T1: After intervention completion	(1) Parental self‐efficacy (PSAM).	(1) Parental self‐efficacy ↑ (intervention: T0 *M* (SD) = 31.40 (6.40), T1 *M* (SD) = 53.87 (13.35); control: T0 *M* (SD) = 30.07 (5.73), T1 *M* (SD) = 29.00 (7.58); between‐group *d* = 2.29 [1.33, 3.25]).	Attendence: 100%. Assessed for eligibility: *N* = not reported. Inclusion criteria met: *N* = 60. EG: T0: *N* = 15; T1: *N* = 15. CG: T0: *N* = 15; T1: *N* = 15.
Baratian et al. (2017) **Iran**	Quasi‐experimental with control group	***N*** = 30 mothers of children with cancer (EG: *N* = 15, CG(NT): *N* = 15) **Age**: EG: *M* (SD) = 30.45 (3.20), CG: *M* (SD) = 29.55 (2.91) **Condition**: Child's cancer **Time of diagnosis**: Any	**Dose**: 120 min, weekly. **Delivery mode**: Group, in‐person. **Provider qualification**: Not reported.	T0: Baseline T1: After intervention completion	(1) Quality of life (WHO‐QOL‐26).	(1) Quality of life ↑ (intervention: T0 *M* (SD) = 70.40 (6.40), T1 *M* (SD) = 92.97 (13.35); control: T0 *M* (SD) = 69.07 (5.73), T1 *M* (SD) = 68.00 (7.58); between‐group *d* = 2.29 [1.37, 3.21]).	Attendence: 100%. Assessed for eligibility: *N* = 52. Inclusion criteria met: *N* = 30. EG: T0: *N* = 15; T1: *N* = 15. CG: T0: *N* = 15; T1: *N* = 15.
Bautista et al. (2023) **Spain**	Randomized nonconcurrent multiple‐baseline SCED	***N*** = 12 parents of a child in active treatment for cancer with medium or high psychosocial risk (measured with PAT) **Age**: *M* (SD) = 41.42 (8.71) **Condition**: Child's cancer (most frequent leukemia) **Time since diagnosis**: At least 1 month from diagnosis	**Dose**: 60 min, weekly. **Delivery mode**: Individual, online. **Provider qualification**: 2‐year postgraduate training in ACT.	Aggregated T0: Repeated measurements before intervention (2‐ or 4‐week baselines, daily ecological measures). Aggregated T1: Measurements during the active intervention. T2: 8‐week follow‐up after completing the second session.	(1) Depression and anxiety (PHQ‐4). (2) Repetitive negative thinking (RNTQ‐3). (3) Valued living (VQ‐3).	(1) Depression and anxiety ↓ (T0 *M* (SD) = 5.07 (3.26), T2 *M* (SD) = 2.60 (1.81); *d* = 0.83 [0.26, 1.40]). (2) Repetitive negative thinking ↓ (T0 *M* (SD) and T2 *M* (SD) not stated; *d* = 0.81 [0.34, 1.28]). (3) Valued living ↑ (T0 *M* (SD) and T2 *M* (SD) not stated; *d* = 1.07 [0.22, 1.91]).	Attendence: 100%. Assessed for eligibility: *N* = 27. Inclusion criteria met: *N* = 16. Discontinued during baseline: *N* = 3. Aggregated T0: *N* = 12. Aggregated T1: *N* = 12. T2: *N* = 12.
Jin et al. (2025) **China**	RCT	***N*** = 142 parents of children with cancer (EG: *N* = 71; CG(TAU): *N* = 71) **Age**: EG: *M* (SD) = 34.99 (5.00), TAU: *M* (SD) = 35.17 (5.92) **Condition**: Child's cancer (91% hematological) **Time since diagnosis**: Within 6 months	**Dose**: 45–60 min, weekly. **Delivery mode**: Individual, first session in person, Sessions 2–4 with synchronous videos. **Provider qualification**: 14 full days ACT skill training and videotaped sessions reviewed by an ACT expert.	T0: baseline T1: 1 week after intervention completion T2: 3 months after T1	(1) Parental psychological distress (DASS‐21). (2) Quality of life (PedsQL Family Impact Module). (3) Parental experience of child's illness (PECI). (4) Experiential avoidance (AAQ‐II). (5) Psychological flexibility (CompACT). (6) Child's psychological adjustment (SDQ).	(1) Parent psychological distress ↓ (intervention: T0 *M* (SD) = 35.18 (24.31), T2 *M* (SD) = 17.72 (17.33); control: T0 *M* (SD) = 28.68 (20.89), T2 *M* (SD) = 22.50 (16.82); between‐group *d* = 0.61 [0.27, 0.94]). (2) QoL ↑ (intervention: T0 *M* (SD) = 56.06 (18.27), T2 *M* (SD) = 69.23 (17.83); control: T0 *M* (SD) = 58.06 (16.36), T2 *M* (SD) = 63.39 (13.86); between‐group *d* = 0.49 [0.16, 0.83]). (3) Guilt and worry ↓ (intervention: T0 *M* (SD) = 2.33 (0.65), T2 *M* (SD) = 1.36 (0.66); control: T0 *M* (SD) = 2.20 (0.71), T2 *M* (SD) = 1.97 (0.72); between‐group *d* = 1.29 [0.93, 1.65]). Unresolved sorrow and anger ↓ (intervention: T0 *M* (SD) = 2.18 (0.79), T2 *M* (SD) = 1.39 (0.77); control: T0 *M* (SD) = 2.12 (0.76), T2 *M* (SD) = 1.88 (0.70); between‐group *d* = 0.71 [0.37, 1.05]); long‐term uncertainty ↓ (intervention: T0 *M* (SD) = 2.01 (0.98), T2 *M* (SD) = 1.20 (0.86); control: T0 *M* (SD) = 2.02 (0.91), T2 *M* (SD) = 1.61 (0.80); between‐group *d* = 0.41 [0.08, 0.75]); emotional resources ↑ (intervention: T0 *M* (SD) = 2.20 (0.87), T2 *M* (SD) = 2.57 (0.94); control: T0 *M* (SD) = 2.37 (0.80), T2 *M* (SD) = 2.36 (0.79); between‐group *d* = 0.53 [0.19, 0.86]). (4) Experiential avoidance ↓ (intervention: T0 *M* (SD) = 25.49 (9.95), T2 *M* (SD) = 18.40 (9.03); control: T0 *M* (SD) = 23.72 (9.43), T2 *M* (SD) = 22.18 (8.11); between‐group *d* = 0.57 [0.24, 0.91]).	Attendence: 80%. Assessed for eligibility: *N* = 250. Inclusion criteria met: *N* = not stated. EG: T0: *N* = 71; T1: *N* = 70; T2: *N* = 65. CG: T0: *N* = 71; T1: *N* = 62; T2: *N* = 56.
						(5) Psychological flexibility ↑ (intervention: T0 *M* (SD) = 61.34 (12.93), T2 *M* (SD) = 69.83 (11.82); control: T0 *M* (SD) = 63.65 (12.07), T2 *M* (S*D*) = 65.23 (11.40); between‐group *d* = 0.63 [0.30, 0.97]). (6) Child's psychological difficulties ↓ (intervention: T0 *M* (SD) = 13.56 (5.34), T2 *M* (SD) = 10.34 (4.91); control: T0 *M* (SD) = 13.04 (5.12), T2 *M* (SD) = 11.93 (5.02); between‐group *d* = 0.39 [0.06, 0.73]).	
Kalateh et al. (2024) **Iran**	Quasi‐experimental with control group	***N*** = 30 mothers of children with cancer (EG: *N* = 15, CG(NT): *N* = 15) **Age**: Only range reported (25–45) **Condition**: Child's cancer **Time of diagnosis**: Any	**Dose**: Not reported, weekly. **Delivery mode**: Group, in‐person. **Provider qualification**: Not reported.	T0: Baseline T1: After intervention completion.T2: Follow‐up (timing not reported)	(1) Identity crisis (ICQ). (2) Distress tolerance (DTS).	(1) Identity crisis ↓ (intervention: T0 *M* (SD) = 166.60 (30.09), T1 *M* (SD) = 114.43 (25.73); control: T0 *M* (SD) = 158.02 (28.21), T1 *M* (SD) = 156.84 (27.42); between‐group *d* = 1.60 [0.77, 2.42]; *η^2^* = 0.66). ^e^ (2) Distress tolerance ↓ (intervention: T0 *M* (SD) = 54.66 (16.17), T1 *M* (SD) = 44.95 (18.14); control: T0 *M* (SD) = 42.96 (19.33), T1 *M* (SD) = 44.03 (16.87); between‐group *d* = 0.05 [−0.66, 0.77]; partial *η^2^* = 0.87). ^ef^	Attendence: 100%. Assessed for eligibility: *N* = not stated. Inclusion criteria met: *N* = not stated. EG: T0: *N* = 15; T1: *N* = 15; T2: *N* = 15. CG: T0: *N* = 15; T1: *N* = 15; T2: *N* = 15.

*Note*: The studies are arranged following the three depicted subpopulations.

Abbreviations: AAQ‐II, Acceptance and Action Questionnaire‐II; CFS, Cognitive Flexibility Scale; CG, control group; CompACT, Comprehensive Assessment of Acceptance and Commitment Therapy Processes; DASS, Depression Anxiety Stress Scales; EG, experimental group; GAD‐7, Generalized Anxiety Disorder Scale‐7; HB, Health Behavior Engagement Questionnaire; PedsQL, Pediatric Model of Health Related Quality of Life; NSSR, Neuropsychological Symptom Self‐Report; NT, no treatment; PAT, Psychosocial Assessment Tool; PCL‐5/C, posttraumatic stress disorder (PTSD)‐5/checklist/compact; PECI, parent experience of child illness; PHQ‐9/4, Patient Health Questionnaire‐9/4; QoL, quality of life; RNTQ‐3, Repetitive Negative Thoughts Questionnaire‐3; SDQ, Strengths and Difficulties Questionnaire; TAU, treatment as usual; VQ‐3, Valuing Questionnaire‐3; WL, waitlist.

^a^Deviation from standard depiction, since means and SDs at T0, T1, and T2 were not reported.

^b^In the demographic table is stated *N* = 9 but then describes the age of 10 participants. In the study is stated *N* = 11 at T0 and *N* = 9 at T1.

^c^Calculated based on T2 sample sizes.

^d^Allocated: *N* = 147, completed T0 survey: *N* = 142.

^e^Control group data at T2 not provided, between group *d* at T2 not measurable.

^f^On the DTS, higher scores indicate greater distress tolerance. The reported descriptive statistics therefore indicate a decrease in distress tolerance in the intervention group.

#### 3.2.1 Participants

A total of 573 people were recruited across the included studies. Of these, 63 participants were AYAs currently diagnosed with cancer or cancer survivors. A further 242 participants were AYAs affected by parental cancer, and 244 were parents of CAYAs affected by cancer. The remaining 24 participants, primarily from Patterson et al. (2022) were siblings (including bereaved siblings) of patients with cancer. Participants of Patterson et al. (2022, 2021) contribute to both the AYAs with cancer or cancer survivors and the AYAs affected by parental cancer categories; the study is nonetheless counted once among the nine included studies.

#### 3.2.2 Intervention

*Delivery characteristics*: Delivery format and intensity varied considerably across studies (see Table 1). All AYA‐adapted programs used a group format to foster peer connection and normalize the cancer experience, with a shared thematic focus on identity reintegration post‐cancer. Notable structural differences included TRUCE's incorporation of a joint parent–child session (Bibby et al. 2024) and the use of peer facilitators alongside professional staff in PEER (Patterson et al. 2022; Patterson et al. 2021) and *Places You'll Go* (Clarke et al. 2021). Both high‐quality parent studies (Bautista et al. 2023; Jin et al. 2025) used individual formats. Provider training ranged from general ACT psychoeducation to postgraduate specialization; treatment fidelity was formally assessed only by Jin et al. (2025) (ACT‐FM (O'Neill et al. 2019); self‐ratings *M* = 4.8–6.7 on a seven‐point scale) and Bautista et al. (2023) (97.2% adherence, 95.8% competent delivery). *Contents*: Specific therapy kernels addressed in the single interventions are depicted in Table 2. All interventions targeted the core processes of ACT. Creative hopelessness was explicitly incorporated in the majority of the intervention's variants (Bibby et al. 2024; Patterson et al. 2022; Baratian et al. 2017; Akbari et al. 2019; Jin et al. 2025), being an important tool to identify experiential avoidance and cognitive fusion. Metaphors were a central delivery mechanism across all studies.

**TABLE 2:** Therapy kernels of encompassed interventions.

Clarke et al. (2021)	Patterson et al. (2022)	Stuart (2022)	Bibby et al. (2024)	Akbari et al. (2019)	Baratian et al. (2017)	Bautista et al. (2023)	Jin et al. (2025)	Kalateh et al. (2024)
Session 1: Psychoeducation on values and goals, introduction to the concept of mindfulness, mindful breathing. Session 2: Understanding one's identity and one's cancer experience, explore different parts of self, including the visible and invisible parts. Changes in values and identity after cancer, introduction of flexible self‐view and meaning making. Session 3: Psychoeducation about acceptance, experiential introduction to defusion and defusion exercises. Session 4: Sharing the own cancer story, action plan on how to share it, psychoeducation on committed action with discussion on the obstacles that might come up. Session 5: Identifying values and goals to focus on after the program, psychoeducation on SMART goals, development of a committed action plan, letter writing activity to a cancer patient to explore the changes in identity and to share learnt coping strategies.	Session 1: Program overview, sharing personal values, impact of values on building stronger supportive relationships, normalizing living with cancer. Session 2: Active sharing and listening of individual cancer experiences. Session 3: Creative hopelessness, mindfulness, acceptance, tug war with a monster, hands as thoughts. Session 4: Challenges of young people affected by cancer, values they've learnt from these challenges, paper lanterns with the values, sharing values. Session 5: Compassion, self‐compassion, self‐kindness, and its barriers, exercises practicing self‐kindness. Session 6: Exploring and practicing the concept of common humanity, impact of having someone else acknowledge their own pain, writing compassionate letter to self about something they tend to judge. Session 7: Introduction and normalization of help‐seeking behavior, reduce barriers to help‐seeking behaviors, brainstorm on how to recognize when additional support is required, check‐in app.	Session 1: Program overview, expectation from participants and metaphors. Session 2: Cognitive defusion, self‐as‐context, experiential acceptance, mindfulness. Session 3: Value‐based living and committed action. Session 4: Experiential avoidance and the processes of the “Inflexahex”. Session 5: The happiness myth, recap of program overview, and wrap up.	Session 1: Introduction to ACT, psychoeducation on the impact of parental cancer, sharing own experiences and normalizing feelings and fears, identifying how people try to control difficult feelings. Session 2: Creative hopelessness, developing a different relationship with feelings (acceptance and mindfulness, committing to a values‐consistent activity). Session 3: Mindfulness, BOLD, noticing when people are acting consistent with their values. Session 4: Mind as a problem‐finding and problem‐solving machine, sticky thoughts (try to push out unwanted thoughts and notice what happens vs. notice but don't try to control). Session 5: Getting unstuck from difficult thoughts, values, and committed action. Session 6: Joint session (young person and parent), values, loving–kindness and self‐compassion, homework: For parents, love–kindness; for young people practice BOLD and self‐compassion. Session 7: Values and goals for the future, action plan, and review.	Session 1: Explaining program structure and treatment goals, introducing ACT concepts, reviewing participants’ history and their children's cancer, creative hopelessness. Session 2: Creative hopelessness, experiential avoidance vs. acceptance, cognitive defusion techniques, metaphors to illustrate ineffectiveness of experiential avoidance, introduction to values. Session 3: Values, chessboard, and bus metaphor, values‐aligned activities, identifying barriers to valued living, mindfulness, introducing self‐as‐context. Session 4: Values and goal‐setting based on values, committed action, discussing barriers to goals and values‐aligned living, mindfulness exercises. Sessions 5–7: Continuing mindfulness practice with decreasing therapist guidance, values‐based goal setting and action planning, exercises to increase satisfaction with values‐aligned living, cognitive defusion exercises, acceptance practice for difficult emotions and experiences. Sessions 8 and 9: Reviewing progress and obstacles, continuing mindfulness and defusion practices, consolidating skills across all six ACT processes. Session 10: Summary and review of entire program, consolidation of learning across all ACT processes, discussion of maintaining gains and continuing practice.	Session 1: Purpose of the session, introduction of ACT and its processes. Session 2: Creative hopelessness, psychoeducation on experiential avoidance, metaphors (man in the well, tug‐of‐war with a monster, hungry tiger). Session 3: Psychoeducation on control strategies with internal events, workability of used strategies. Session 4: Teaching acceptance as an alternative (analogy of a lion, lion, lion), relaxation techniques. Session 5: Cognitive defusion (metaphors of the bus and marching army), mindfulness. Session 6: Self‐as‐context (chess metaphor, house with furniture), learning to see emotions as consequence of a context. Session 7: Values, motivation to change, practicing your own funeral, values assessment rating. Session 8: Committed action, action plans in accordance with values, difference between desires and wants (analogy of the beggar at the door), evaluation of the intervention.	Session 1: Presentation of the intervention aim: focus on valued actions and disrupt RNT, exploring RNT as a central psychopathological process and exploring its triggers and how valued actions reduce RNT, exploration of experiential avoidance strategies linked to RNT, introduction of an experiential metaphor in the consequences of RNT and value‐based living. Session 2: Exercises to promote discrimination of the RNT process and to promote engaging in valued actions, introduction of audio files with exercises to promote practicing the skills trained.	Session 1: Generation of creative hopelessness, Chinese finger trap metaphor, passengers on the bus, mindful exercises, and openness to your experiences as homework. Session 2: Observing your thoughts, thoughts‐on‐cards exercise, floating leaves on a moving stream exercise, homework assignment (“Unhook from your thoughts/mind”). Session 3: Mindful sitting, mindful eating, “the story line” exercise, defusing from self‐concept (the good parent/bad parent exercise). Session 4: Mindful body scan, value clarification, two kids in car heading to an amusement park metaphor, value‐based action plan.	Session 1: Getting to know the goals of therapy (telling the “pain story” of each member in his own language and conceptualizing the problem), examining the client's experiential avoidance, and creating creative frustration with the metaphors of the yellow jeep, the ball pool, and the man in the well. Session 2: Explaining the concept of acceptance using the metaphors of Jack the Tramp, the baby on the plane, the exercise of compassion, the yo‐yo metaphor, and the metaphor of the house on the side of the road. Session 3: Teaching how to use the metaphors of leaves floating in the river and thoughts floating in the room and practicing hands as thoughts, the metaphor of bus passengers and the metaphor of pickles. Session 4: Training on the use of popular news hour metaphors, practice of good moments with open and closed eyes, practice of writing autobiography, and 10 steps to achieve values and scoreboard metaphor. Session 5: Teaching the use of metaphors like chess board, watching TV and self‐conceptualized trial, and the prince and beggar. Session 6: Training to use mindful walking exercises, eating raisins, dandelion practice, mindful breathing, practicing awareness of experiences. Session 7: Training and metaphor of bubbles and movement on the road and bicycle factory. Session 8: A blueprint for the future, reviewing committed action as a “lifetime commitment.”

*Note*: The studies are arranged following the three depicted subpopulations.

Abbreviations: BOLD, breathing, observing, listening, and deciding; RNT, repetitive negative thinking; SMART, specific, meaningful, adaptive, realistic, time‐framed.

Every protocol included psychoeducation and exercises on values and committed action, often linked to value‐driven goal setting (Bibby et al. 2024; Jin et al. 2025). Acceptance was explicitly integrated in eight curricula, whereas compassion was explicitly integrated in three (Bibby et al. 2024; Patterson et al. 2022; Kalateh et al. 2024).

Different in intervention adaptations for AYAs was the use of the process of self‐as‐context as a way of identity reintegration (Clarke et al. 2021). Additionally, there was a general focus on active learning methods (e.g., tinkering with paper lanterns with values) and on peer interactions.

In interventions for parents, there is an emphasis on classic verbally complex ACT metaphors and skill training to better support their children.

#### 3.2.3 Outcome Measures

The outcome measures were psychological outcomes and health‐related QoL. The assessment timepoints varied broadly in the nine studies. Three studies evaluated the outcomes only once pre‐ and postintervention. The other five studies used multiple timepoints to assess follow‐up effects, including 2 (Bibby et al. 2024; Patterson et al. 2022; Bautista et al. 2023) and 3 months of follow‐up (Jin et al. 2025). Kalateh et al. (2024) didn't report how long the follow‐up was.

The range of intervention attendance reported across the included studies is 80%–100%.

### 3.3 Risk of Bias in Studies

Tables 3 and 4 report the risk of bias ratings for the RCT, the nonrandomized controlled studies, and the single group pre‐post evaluations. The complete assessment for all eight studies is provided in the OSF Project. The study by Clarke et al. (2021) was not assessed for risk of bias, as it solely reported the acceptability and feasibility of the intervention.

**TABLE 3:** Risk of bias through JBI critical appraisal checklist for nonrandomized experimental studies.

	Kalateh et al. (2024)	Baratian et al. (2017)	Bibby et al. (2024)	Patterson et al. (2022)	Akbari et al. (2019)	Stuart (2022)
1. It is clear in the study what is the “cause” and what is the “effect” (i.e., there is no confusion about which variable comes first)?	√	√	√	√	√	√
2. Was there a control group?	√	√	√	x	√	x
Were participants included in any comparisons similar?	x	?	x	—	?	—
4. Were the participants included in any comparisons receiving similar treatment/care, other than the exposure or intervention of interest?	?	?	√	—	?	—
5. Were there multiple measurements of the outcome, both pre and post the intervention/exposure?	x	x	x	x	x	x
6. Were the outcomes of participants included in any comparisons measured in the same way?	√	√	√	—	√	—
7. Were outcomes measured in a reliable way?	√	√	√	?	√	√
8. Was follow‐up complete and if not, were differences between groups in terms of their follow‐up adequately described and analyzed?	x	√	?	x	√	x
9. Was appropriate statistical analysis used?	?	?	√	√	?	√
Overall risk^a^	HIGH	HIGH	HIGH	HIGH	HIGH	HIGH

*Note*: “ √ ” = yes; “x” = No; “?” = not clear; “‐” = not applicable. Critical bias domains: Questions 2, 7, 8, and 9.

^a^HIGH: x in a critical bias domain; MODERATE: more than three x or ? in noncritical domains; LOW: less or equal to three x or ? in noncritical domains.

**TABLE 4:** Risk of bias through JBI critical appraisal checklist for randomized experimental studies.

	Jin et al. (2025)
1. Was true randomization used for assignment of participants to treatment groups?	√
2. Was allocation to treatment groups concealed?	√
3. Were treatment groups similar at the baseline?	√
4. Were participants blind to treatment assignment?	x
5. Were those delivering the treatment blind to treatment assignment?	x
6. Were treatment groups treated identically other than the intervention of interest?	√
7. Were outcome assessors blind to treatment assignment?	x
8. Were outcomes measured in the same way for treatment groups?	√
9. Were outcomes measured in a reliable way?	√
10. Was follow‐up complete and if not, were differences between groups in terms of their follow‐up adequately described and analyzed?	√
11. Were participants analyzed in the groups to which they were randomized?	√
12. Was appropriate statistical analysis used?	√
13. Was the trial design appropriate and any deviations from the standard RCT design (individual randomization, parallel groups) accounted for in the conduct and analysis of the trial?	√
Overall risk^a^	LOW

*Note*: “ √ ” = yes; “x” = No; “?” = not clear; “‐” = not applicable. Critical bias domains: Questions 1, 2, 3, 9, and 10.

^a^HIGH: x in a critical bias domain; MODERATE: more than three x or ? in noncritical domains; LOW: less or equal to three x or ? in noncritical domains.

Inter‐rater reliability was high across the assessed tools. Agreement was 94.4% for the six studies assessed with the JBI nonrandomized checklist and 92.3% for the single RCT. For the single‐case experimental design assessed with the RoBiNT scale, agreement was 93.3%.

All nonrandomized and pre‐post studies were rated as high overall risk of bias, primarily due to selection and allocation concerns: absence of baseline equivalence testing (Bibby et al. 2024; Baratian et al. 2017; Akbari et al. 2019; Kalateh et al. 2024), geographic rather than random allocation (Bibby et al. 2024), and implausibly large effect sizes (*d* > 2.00) suggesting measurement bias (Baratian et al. 2017; Akbari et al. 2019). Stuart (2022) was additionally limited by severe underpowering (22% of required sample) and a 5.4% participation rate. Patterson et al. (2022) used a non‐validated primary outcome measure. Jin et al. (2025) and Bautista et al. (2023) were rated low risk of bias.

### 3.4 Synthesis of Findings With Certainty of Evidence

The synthesis of findings is structured by population group as depicted in the PICO criteria: AYAs in cancer treatment or who survived cancer, AYAs impacted by parental cancer, and parents of CAYAs with cancer.

Due to the small number of studies included for each outcome (< 10), formal tests for funnel plot asymmetry were not conducted. Consequently, the risk of publication bias could not be statistically assessed.

#### 3.4.1 ACT for CAYAs With Cancer and for Cancer Survivors

Three studies examined ACT in AYAs with cancer or survivors (Stuart 2022; Patterson et al. 2022; Clarke et al. 2021), though in practice only 3.3% of participants in Patterson et al.’s (2022, 2021) PEER program were on active treatment (12.5% were cancer survivors, the remainder were siblings or offspring of a person with cancer). No study specifically investigated ACT for children under 12.

Regarding direct clinical effectiveness, Stuart (2022) found no significant effect across all outcomes in a severely underpowered pre‐post study (*N* = 11; 22% of required sample) at high risk of bias; these findings may reflect the study's inability to detect an effect if one existed. Patterson et al. (2022, 2021), also using an uncontrolled design, reported that approximately 20% of participants experienced clinically meaningful QoL improvements, but the absence of mean‐level data precluded effect size calculation.

Regarding mediation findings, more informative was Patterson et al.’s (2022) mechanistic analysis of 52 highly distressed AYAs (with cancer/cancer survivors or affected by parental cancer), in which QoL improvements were significantly mediated by psychological flexibility *(β =* 0.21, SE = 0.11) and self‐kindness (*β* = 0.26, SE = 0.12), but not by general peer support or mindfulness. Acceptability was high: 85% of AYAs in Clarke et al. (2021) rated sessions as helpful and 89% as meaningful.

Certainty of evidence for psychosocial outcomes was rated very low (⊕⊝⊝⊝), reflecting uncontrolled designs, small samples (*N* = 63), and high risk of bias.

#### 3.4.2 ACT for AYAs Impacted by Parental Cancer

Two studies evaluated ACT for AYAs with parents with cancer: Bibby et al. (2024) (*N* = 142, pragmatic controlled trial) and Patterson et al. (2022), in which 44 of 52 distressed participants (85%) were siblings or offspring of a person with cancer, including 13 (25%) who were bereaved.

Findings were inconsistent across outcomes. Bibby et al. (2024) (a controlled trial with nonrandom allocation) found no significant between‐group differences in psychological distress or psychological flexibility at follow‐up, whereas Patterson et al. (2022) (an uncontrolled pre‐post study) reported significant reductions in distress. Bibby et al. (2024) did not measure QoL directly but found a significantly greater reduction in unmet needs (a construct closely related to psychosocial well‐being in this population (Patterson et al. 2017)) in the intervention group than in controls (time × condition interaction, *d* = 0.29). Because of the limited and heterogenous evidence, final conclusions about effectiveness cannot yet be drawn.

As previously noted in Section 3.4.1, mediation analyses within this same distressed subsample identified psychological flexibility as a significant mediator of change in Patterson et al. (2022).

Certainty of evidence was rated very low (⊕⊝⊝⊝) across all outcomes, due to inconsistency between studies, nonrandom allocation, and absence of controlled designs for the Patterson et al. (2022) distressed subsample.

#### 3.4.3 ACT for Parents of CAYAs With Cancer

Five studies examined ACT for parents: one RCT (Jin et al. 2025) and one single case experimental design (SCED) (Bautista et al. 2023) with low risk of bias, and three quasi‐experimental designs (Baratian et al. 2017; Akbari et al. 2019; Kalateh et al. 2024) with high risk of bias. Because the other strata presented uniformly very low certainty, SoF tables was exclusively generated for this group (Table 5).

**TABLE 5:** Synthesis of findings table for critical outcomes in studies with parents of CAYAs with cancer.

Outcome domain	Effect size range (*d*)	No. of studies (design)	GRADE assessment	Key findings and comments
Symptoms of mental disorders (distress, depression, anxiety)	*d* = 0.61–0.83	2 (1 RCT, 1 SCED)	⊕⊕⊕⊝ Moderate	– Consistent medium‐to‐large improvements in high‐quality studies (Jin et al. 2025; Bautista et al. 2023). – Downgraded once for imprecision (small *k*)
Quality of life	*d* = 0.49–2.29	2 (1 RCT, 1 quasi‐experimental)	⊕⊝⊝⊝ Very low	– Jin et al. (2025) showed medium effect (*d* = 0.49). – Baratian et al. (2017) reported implausibly large effect (*d* = 2.29). – Downgraded for serious inconsistency risk of bias and imprecision.
Psychological flexibility	*d* = 0.63	1 (RCT)	⊕⊕⊕⊝ Moderate	– Significant increase in flexible behaviors (Jin et al. 2025). – Downgraded for imprecision (single study).

Abbreviation: SCED, single case experimental design.

Symptoms of mental disorders were assessed by Jin et al. (2025) in their RCT and Bautista et al. (2023) in their SCED, who reported medium‐to‐large effects on distress and anxiety (*d* = 0.61 and *d* = 0.83, respectively). Certainty was rated moderate (⊕⊕⊕⊝), downgraded once for imprecision (*k* = 2).

QoL was assessed in their RCT by Jin et al. (2025) (*d* = 0.49) and in their quasi‐experimental study with high risk of bias by Baratian et al. (2017) (*d* = 2.29). The implausibly large effect in Baratian et al. (2017), likely reflecting baseline imbalances and measurement bias, introduced serious inconsistency. Certainty was rated very low (⊕⊝⊝⊝).

Psychological flexibility was assessed directly only by Jin et al. (2025) (RCT), who reported significant increases (*d* = 0.63; moderate certainty, ⊕⊕⊕⊝; downgraded for imprecision, *k* = 1).

Other ACT and non‐ACT–specific process outcomes showed large effects: reductions of RNT (*d* = 0.81), more valued living (*d* = 1.07) in Bautista et al. (2023) (SCED), identity crisis (*d* = 1.60) (Kalateh et al. 2024) and increases in parental self‐efficacy (*d* = 2.29) (Akbari et al. 2019)—the latter two deriving from quasi‐experimental studies at high risk of bias. Kalateh et al. (2024) additionally assessed distress tolerance (DTS), but the direction of this effect could not be established: The reported means indicate a decrease in DTS in the intervention group with no between‐group difference at posttest (*d* = 0.05 [−0.66, 0.77]), whereas the authors report a significant increase in DTS (*F* = 181.34, *p* = 0.001, partial *η^2^* = 0.87). In view of this discrepancy, we do not interpret this outcome. Finally, Jin et al. (2025) (RCT) reported a small but statistically significant effect on children‘s psychological adjustment (Strengths and Difficulties Questionnaire [SDQ]; *d* = 0.39).

## 4 Discussion

### 4.1 Summary of Main Findings

This systematic review aimed to evaluate the acceptability, feasibility, and efficacy of ACT within three interconnected population strata: CAYAs with cancer or cancer survivors, CAYAs impacted by parental cancer, and parents of CAYAs affected by cancer. While efficacy was evaluated through standardized clinical outcomes and appraised via GRADE, acceptability was evidenced by consistently high session attendance rates (exceeding 80%) and zero reported adverse events across the included trials. The review identified nine studies involving 573 participants.

For AYAs, the evidence base was heterogeneous and characterized by high risk of bias, precluding firm conclusions about ACT's effectiveness in this population. Preliminary findings suggested a possible benefit for psychological flexibility (Patterson et al. 2022), though effects on general distress were not consistently detected across the available studies. Critically, no studies were found that adapted ACT for children undergoing cancer treatment or cancer survivors (< 12 years), highlighting a significant gap in the literature.

ACT for parents showed medium‐to‐large effects on distress, anxiety, depression, and psychological flexibility in higher‐quality studies (Bautista et al. 2023; Jin et al. 2025), and very large effects in studies with high risk of bias (Baratian et al. 2017; Akbari et al. 2019; Kalateh et al. 2024) that should be interpreted with caution.

### 4.2 Interpretation in Context

Across strata, findings can be linked back to ACT's theoretical framework. Although no included study designated symptom reduction as its primary target, moderate‐certainty evidence for parents showed improvements in parental psychological distress (*d* = 0.61) (Jin et al. 2025) and in depressive and anxiety symptoms (*d* = 0.83) (Bautista et al. 2023).

Moreover, parent samples were not homogeneous in symptomatology, but rather in the shared experience of a child's somatic illness, which can elicit heterogeneous forms of psychopathology across caregivers. Despite this diagnostic heterogeneity, interventions structured around ACT's general processes of change (Jin et al. 2025) and those structured around a functional diagnosis (Hayes et al. 1996) targeting RNT (Bautista et al. 2023), both yielded improvements across ACT‐specific and non‐ACT‐specific measures, including gains in children's psychological adjustment. These findings might support ACT's claim of transdiagnostic efficacy and the relevance of psychological flexibility for both parents and their children (Leeming and Hayes 2016).

While evidence for parents showed moderate certainty, findings for AYAs were inconclusive or of low certainty. Four arguments might account for this discrepancy: delivery mode (individual vs. group), the selection of outcome measures (proximal vs. distal), and the reliance of adolescent ACT manuals on unmodified derivatives of standardized adult protocols, and lower methodological robustness (see Section 4.3).

#### 4.2.1 Interpreting the Parent–AYA Discrepancy

One explanation advanced for the moderate‐to‐strong effects in parent studies (Bautista et al. 2023; Jin et al. 2025) is their individual delivery format, which permits closer tailoring to each participant's context. We raise this only as a candidate explanation, as it cannot be tested within this evidence base (see Section 4.2.2). A related and equally untested hypothesis is that the group format used in all AYA interventions afforded fewer opportunities for individualized case conceptualization, as diagnosis‐homogeneous groups can allow “typical” cancer‐related themes to dominate discourse over participants’ own values and goals.

Furthermore, the largely null or inconsistent findings across the AYA trials (Bibby et al. 2024; Stuart 2022; Patterson et al. 2022) may in part be an artifact of measurement selection rather than a lack of efficacy. Many studies relied on distal, broad constructs like global QoL, which are influenced by numerous nonpsychological factors (e.g., physical side effects, financial strain) and may be insensitive to short‐term therapeutic changes. More proximal measures that map directly onto ACT processes or specific distress markers in psycho‐oncology may be more sensitive, as ACT explicitly targets those as the mechanism of change. For instance, Bautista et al. (2023) successfully detected changes by assessing RNT—a construct closely tied to cognitive fusion and experiential avoidance—and valued living, detecting significant reductions (RNT: *d* = 0.81; valued living: *d* = 1.07). This illustrates how theoretically aligned, process‐specific measures can reveal change that a distal outcome like global QoL might miss. This pattern suggests that evaluations of ACT for AYAs may benefit from prioritizing process measures (e.g., psychological flexibility, cognitive fusion, valued living) as primary outcomes, with distal functional outcomes treated as secondary, longer‐term indicators consistent with ACT's own theory of change.

Another critical factor contributing to the nonsignificant AYA findings may be a fundamental misalignment between the interventions and adolescent developmental stages. Although included studies utilized specialists and adolescent client feedback to inform their designs (e.g., 66) and generally changed their interventions to better suit AYAs’ needs, the resulting protocols still largely relied on adapting downward from adult manuals (e.g., the *Take‐A‐Breath* protocol (Burke et al. 2014; Rayner et al. 2016) adapted by Stuart (2022) for young cancer survivors) or simplifying general ACT metaphors to be more playful. Adapting adult modules implicitly treats the adult mind as the normative baseline (Hayes et al. 2020), failing to be culturally responsive (Ong and Lee 2025) and risking a failure to capture the distinct biopsychosocial maturational profiles that characterize adolescence.

Despite these limitations, the included studies did make important structural efforts to accommodate young patients. In the AYA studies, group‐based formats were used to foster peer connection and normalize the cancer experience, while more experiential and playful exercises were incorporated to better fit developmental needs and participants’ energy levels (Bibby et al. 2024; Stuart 2022; Patterson et al. 2022; Clarke et al. 2021).

#### 4.2.2 Confounding Moderators Across the Included Trials

The four arguments developed above cannot be weighed in isolation. The included trials differ systematically in who was recruited, in how the interventions were delivered, and in how change was measured, and each of these domains offers an alternative account of the attenuated effects observed in the group trials.

First, recruitment and participant characteristics could plausibly moderate the observed effects. These include recruitment strategy and baseline clinical status—the parent trials selected for distress or its proxies (Bautista et al. 2023; Jin et al. 2025), whereas the AYA studies enrolled irrespective of symptom level (Bibby et al. 2024; Patterson et al. 2022)—as well as sample heterogeneity (Patterson et al. 2022), age range (12–17 (Patterson et al. 2022), 14–22 (Bibby et al. 2024), 18–25 (Stuart 2022)), handling of comorbidity and co‐intervention (Bibby et al. 2024; Jin et al. 2025), and prior therapy history, which only one trial recorded (Bibby et al. 2024). Where such factors were examined, they operated in the same direction: Improvements were larger among participants with higher baseline distress and lower baseline mindfulness or psychological flexibility (Bibby et al. 2024; Patterson et al. 2022; Patterson et al. 2021). The recruitment in AYA trials irrespective of symptom level may understate gains in the subgroup with most room to improve.

Second, implementation parameters differed systematically between the individual and group trials: cumulative dose (2–14 h) (Bibby et al. 2024; Bautista et al. 2023), group size (3–10 adolescents with two facilitators vs. a single cohort of 11; Stuart, 2022, Patterson et al., 2022), program span (3–4 residential days (Patterson et al. 2022; Clarke et al. 2021) vs. weekly sessions over 4–7 weeks (Bibby et al. 2024; Stuart 2022; Jin et al. 2025)), and provider preparation, with formal ACT credentials and documented fidelity confined to the individual trials (Bautista et al. 2023; Jin et al. 2025) (Section 3.2.2).

Lastly, measurement varied across the included trials. Construct coverage barely overlaps between studies (Bibby et al. 2024; Stuart 2022; Patterson et al. 2022; Clarke et al. 2021), and assessment schedules diverge, from repeated measurements across several timepoints (Bautista et al. 2023) and pre‐post designs with a 2–3‐month follow‐up (Bibby et al. 2024; Jin et al. 2025) to a single pre‐post comparison (Stuart 2022) or session level ratings only (Clarke et al. 2021). All AYA outcomes were subjective self‐reports, whose reliability may vary across the developmental range covered here, and no trial reported behavioral or functional endpoints such as school attendance or return to valued activity. With baseline severity, dose, provider expertise, fidelity, and measurement all covarying with delivery format, the comparative effectiveness of individual versus group ACT cannot be isolated here; the format hypothesis (Section 4.2.1) is a question for future trials, not a finding of this review.

### 4.3 Limitations of the Evidence

Several limitations of the included studies must be acknowledged. First, methodological quality was highly variable, ranging from robust RCTs to uncontrolled pre‐post designs with high risks of bias (e.g., small sample sizes). Second, the heterogeneity of the populations within each stratum complicates the generalization of findings. Third, reporting quality was a further constraint: In the quasi‐experimental studies with parents (Baratian et al. 2017; Akbari et al. 2019; Kalateh et al. 2024) the reported inferential statistics could not always be reconciled with the accompanying descriptive data—most clearly in Kalateh et al. (2024), whose group means indicate a decrease in DTS while the text reports a significant increase. For these studies, effect direction could not be verified independently of the author's narrative. A distinct and more serious concern applies to Baratian et al. (2017) and Akbari et al. (2019), whose reported means differ by exactly 39.00 in three of the four cells and by 39.10 in the fourth, with all four standard deviations identical. These datasets cannot be treated as independent sources of evidence, although they use different constructs.

Few studies utilized process‐specific measures consistently, making it difficult to confirm if changes were indeed mediated by increased psychological flexibility or by nonspecific therapeutic factors, such as group support. Furthermore, there is a notable absence of health‐related and behavioral outcomes (e.g., sleep, school absenteeism, pain, fatigue). This is a critical gap, as ACT also aims to foster committed action, and thus observable behavioral variation, rather than solely symptom reduction (Hayes et al. 2006). Additionally, no data on the economic viability or cost‐effectiveness of these interventions were reported. Given that many families travel long distances for treatment, brief or remote interventions (e.g., focused ACT) could offer logistical advantages, yet their feasibility remains unexplored in this context.

A critical tension identified in this evidence base is the trade‐off between reproducibility and flexibility. While rigorous trials require standardized manuals to ensure internal validity, ACT is—by virtue of its functional contextualist core—inherently idiographic (Hayes 2004; Hayes 2015). Rigid adherence to a fixed protocol may inadvertently constrain the therapist's ability to conduct real‐moment functional analysis and to “dance around the hexaflex” (Harris 2019).

Finally, treatment fidelity was assessed through fidelity measures in only two of the included studies (Bautista et al. 2023; Jin et al. 2025). This lack of fidelity monitoring represents a significant gap; without it, it is difficult to determine if null results are due to the intervention's failure or a failure to deliver the intervention as intended (O'Neill et al. 2019).

Beyond being reported as a limitation, the heterogeneity observed across strata can also be leveraged analytically. First, delivery format differed systematically by stratum: AYA‐focused interventions consistently used group or camp‐based formats to foster peer connection (Bibby et al. 2024; Stuart 2022; Patterson et al. 2022; Clarke et al. 2021), whereas the two highest‐certainty parent studies used individual delivery (Bautista et al. 2023; Jin et al. 2025). Second, assessment strategy varied in ways that may affect the sensitivity of detected change: Bautista et al. (2023) incorporated ecological momentary assessment, in contrast to the single pre/post timepoints used in all other included studies, which may capture within‐person variability that traditional pre/post designs cannot. Third, the near‐total nonoverlap in outcome measures across strata (e.g., Acceptance and Action Questionnaire‐II [AAQ‐II], CompACT for parents vs. AFQ‐Y8 for AYAs) precludes any direct cross‐stratum comparison of effect magnitude, meaning that observed differences in certainty or effect size may partly reflect measurement choice rather than genuine differences in treatment response. Finally, heterogeneity also arises from unequal population representation within the evidence base: Of 573 total participants, only 63 were AYAs with their own cancer diagnosis, compared with 242 AYAs affected by parental cancer and 244 parents, and Patterson et al.’s (2022) sample itself combined both AYA subgroups, which may obscure subgroup‐specific effects within a single study. Taken together, these four sources of heterogeneity suggest that observed differences in evidence certainty between strata may be driven as much by methodological divergence as by true differences in intervention effectiveness because of the contextual and developmental adjustments in the treatment protocols or the responsiveness of different populations.

### 4.4 Review Limitations

Several limitations of the review process itself warrant consideration when interpreting these findings. First, the definition of the target population involved certain arbitrary boundaries, particularly regarding CAYAs affected by parental cancer. Inclusion was restricted to CAYAs whose parents were currently undergoing cancer treatment, thereby excluding those whose parents were cancer survivors. This distinction is arguably artificial, as the psychological impact of parental cancer on children may often persist long into the survivorship phase (e.g., fear of recurrence, altered family dynamics). The heterogeneity of the included populations complicates the generalizability of the findings. The included populations differ fundamentally in stressor type, clinical context, intervention targets and delivery constraints, outcome priorities, and plausible mechanisms of change. Although grouping these populations may be clinically useful for practitioners working with the entire family system, such heterogeneity substantially limits the validity of any unified conclusions.

Second, methodological deviations from the original protocol were necessary but may have introduced potential bias. The initial plan to use ROBINS‐I V2 was adapted to use JBI tools to better accommodate the high prevalence of single‐group designs. While the JBI tools are rigorous, they lack a standardized algorithm for converting item scores into categorial risk of bias ratings. Consequently, the authors applied a subjective scoring threshold to determine these ratings, which may have influenced the risk of bias classification. Additionally, the GRADE assessment for AYAs was modified to aggregate psychosocial outcomes due to the scarcity of data, which differs from the approach originally planned.​ Finally, language bias couldn't be ruled totally out. Although the review included non‐English studies (e.g., Farsi), these were translated using neural machine translation (DeepL) (DeepL Translator n.d.). While this technology is advanced, subtle nuances in psychotherapeutic terminology or cultural context may have been lost or misinterpreted during extraction.

A potential additional limitation concerns the use of general‐purpose large language models (LLMs) to refine search strings and cross‐check data extraction. Therefore, the use of LLMs for search string refinement and extraction cross‐checking is transparently reported; reproducibility risks are mitigated by the human‐in‐the‐loop approach, with LLMs used exclusively as a secondary audit tool.

### 4.5 Clinical Implications and Future Research Priorities

Clinically, moderate‐certainty evidence supports offering ACT in individual settings to parents of children with cancer to reduce distress, anxiety, depressive symptoms, and repetitive negative thinking and to increase psychological flexibility; the trials supplying this evidence used brief individual formats, which is the form with direct empirical support rather than necessarily the only effective one. For AYAs, while evidence is preliminary, mediation findings suggest psychological flexibility is a process of change. Future clinical adaptation of ACT for CAYAs should explicitly balance standardization with the flexibility required for ACT's idiographic case conceptualization. To address the limited specialized training among practitioners, brief, manualized protocols are essential. Hybrid delivery models (face‐to‐face initiation with digital support) offer a clinically viable strategy to maintain therapeutic alliance while reducing logistical burdens for families.

These findings translate into different research priorities across strata. In the AYAs strata, the included trials recruited universally and pooled participants across wide age bands that span the distinct developmental and dispositional response signatures (see 1.4). Future trials should sample within narrower developmental windows or prespecify age‐stratified analyses (in order to further tailor therapy protocols to developmental and contextual needs), and should consider enrolling above a distress or inflexibility threshold, given that participants with higher baseline psychological inflexibility benefited most (Patterson et al. 2022). Developmentally validated process measures should be complemented by behavioral indicators such as school attendance or activity reengagement, which are largely absent from the current evidence and less dependent on the introspective demands of self‐report (as stated by Recommendation 9 of the ACBS Task Force for advancing contextual behavioral science (Hayes et al. 2021)). Provider training and supervision should be controlled for to exclude quality of delivery as a confounding variable.

Two further priorities follow directly from the developmental considerations and remain unaddressed in the current evidence base. First, the referral pathway was not systematically reported, although it bears on baseline treatment motivation and plausibly differentiates the AYA strata and parent stratum. Second, no trial verified that the processes it targeted were equally accessible and comprehensible across the age range it enrolled; because processes of change must be specified with developmental differences in mind, therapy kernels should be examined inductively and through experimental analogs across that range (Hayes et al. 2021), so that “individual treatment components, rather than broad treatment” packages can be developed and tested. These individual treatment components might benefit from utilizing developmental frameworks like DNA‐V (Hayes and Ciarrochi 2015), which are designed specifically around how youth navigate their evolving ecologies.

No included trial treated a child with cancer under the age of 12. The developmental adaptations outlined in Section 1.4 are therefore untested and in the age group where parent‐mediated delivery would be the most plausible route. Feasibility work using age‐appropriate metaphors, experiential and creative exercises, explicit generalization, and psychoeducation is a precondition for efficacy trials in this group.

For parents, the priorities differ. Brief individual and remote protocols already have moderate‐certainty support, so the principal gaps are replication against active comparators, cost‐effectiveness data, and dyadic outcomes capturing the transmission to child adjustment that the model predicts and that only one included trial assessed (Jin et al. 2025). Across both strata, SCEDs and ecological momentary assessment offer feasible alternatives where recruitment is constrained and idiographic variation is expected, and consistent reporting of cumulative dose, session length, group size, and provider qualification would allow future syntheses to compare across modalities.

## 5 Conclusion

Across nine studies (*N* = 573), ACT is consistently acceptable in the cohorts included in this review but unevenly evidenced. Significant results at moderate certainty were shown only in the parent stratum, where ACT was delivered individually to parents. Small, at high risk of bias and of very low certainty outside two studies, and too heterogeneous to pool, the corpus supports one narrow clinical recommendation and a broad research agenda.

The nine studies span three cohorts: (1) AYAs with cancer or cancer survivors (*k* = 3), (2) AYAs impacted by parental cancer (*k* = 2), and (3) parents of CAYAs affected by cancer (*k* = 5), with Patterson et al. (2022) contributing to two (15.8% with cancer or cancer survivors). The study designs comprised one RCT, one SCED, four quasi‐experimental studies, two single‐group pre‐post studies and one feasibility study. Risk of bias was high in six of the eight appraised studies principally for untested baseline equivalence, nonrandom allocation, and unvalidated measurement.

The individual parent protocols yielded medium‐to‐large effects on psychological distress and anxiety (*d* = 0.61–0.83) (Bautista et al. 2023; Jin et al. 2025) and on psychological flexibility (*d* = 0.63) (Jin et al. 2025), whereas group protocols for parents yielded significant effects at magnitudes (up to *d* = 2.29) (Baratian et al. 2017; Akbari et al. 2019; Kalateh et al. 2024) better explained by design and measurement bias than by treatment. Effects in AYA trials (only group format) were null on nearly all outcomes apart from unmet needs (Bibby et al. 2024).

Attendance was 80%–100% where reported, with no adverse events. As regards external validity, translation extends only to the two brief individual parent protocols (Bautista et al. 2023; Jin et al. 2025) (also because therapy protocols are the best documented), and is bounded elsewhere by mixed samples, logistically demanding formats (e.g., camp‐based), fidelity assessed without standardized measures, and the absence of cost‐effectiveness data.

Research priorities differ by stratum. For AYAs, they are well‐powered studies with narrower developmental windows or age‐stratified analyses, recruitment by baseline distress or psychological inflexibility, developmentally validated and proximal process measures as primary endpoints together with behavioral variables that transcend self‐report limitations, and control for provider qualification and treatment fidelity. Below age 12, feasibility work is a precondition for advancing the research field and extending the reach of ACT. For parents, research priorities are active comparators, more RCTs to extend the certainty of evidence (currently downgraded to moderate for imprecision), cost‐effectiveness data, and dyadic outcomes.

ACT has been supported where it was tested closest to best methodological practice. These conditions have so far been met only in the parent stratum. Testing it on comparable terms in CAYAs will require protocols built from youth‐specific developmental frameworks, and idiographic methods such as SCEDs and ecological momentary assessment where recruitment is constrained.

## Author Contributions

**Giorgio Alagna**: conceptualization, investigation, Writing – original draft, methodology, Writing – review and editing, project administration. **Kim Prox**: methodology, writing – review and editing, investigation, writing – original draft. **Charlotte Hanisch**: conceptualization, writing – original draft, writing – review and editing, methodology, supervision, investigation.

## Funding

The authors have nothing to report.

## Disclosure

The review was preregistered on PROSPERO (registration ID: CRD420251104115). No excerpts from copyrighted works owned by third parties are included.

## Ethics Statement

This study is a systematic review of existing literature; therefore, no ethical approval or informed consent was sought.

## Conflicts of Interest

Giorgio Alagna receives financial support from the Friedrich Ebert Foundation. The other authors declare no conflicts of interest.

## Supporting information




**Supplementary Table**: brb371902‐sup‐0001‐TableS1.docx

**Supporting Information**: brb371902‐sup‐0002‐SuppMat.pdf

## Data Availability

The data that support the findings of this study are openly available in the Open Science Framework (OSF) Project at: https://osf.io/qd6×7.
