# Supplementary material for: Acceptance and Commitment Therapy for Children, Adolescents, and Young Adults With Cancer and Their Parents and for Children, Adolescents, and Young Adults Affected by Parental Cancer: A Population‐Stratified Systematic Review
Source: Brain Behav. 2026 Nov 9;16(11):e71902. doi: 10.1002/brb3.71902 (PMC13746990; doi:10.1002/brb3.71902)
Supplement: Supplementary file 1 [file BRB3-16-e71902-s002.pdf]

PRISMA 2020 for Abstracts Checklist

Section and Topic	Item #	Checklist item	Reported (Yes/No)
**TITLE**			
Title	1	Identify the report as a systematic review.	Yes
**BACKGROUND**			
Objectives	2	Provide an explicit statement of the main objective(s) or question(s) the review addresses.	Yes
**METHODS**			
Eligibility criteria	3	Specify the inclusion and exclusion criteria for the review.	Yes
Information sources	4	Specify the information sources (e.g. databases, registers) used to identify studies and the date when each was last searched.	Yes
Risk of bias	5	Specify the methods used to assess risk of bias in the included studies.	Yes
Synthesis of results	6	Specify the methods used to present and synthesise results.	Yes
**RESULTS**			
Included studies	7	Give the total number of included studies and participants and summarise relevant characteristics of studies.	Yes
Synthesis of results	8	Present results for main outcomes, preferably indicating the number of included studies and participants for each. If meta-analysis was done, report the summary estimate and confidence/credible interval. If comparing groups, indicate the direction of the effect (i.e. which group is favoured).	Yes
**DISCUSSION**			
Limitations of evidence	9	Provide a brief summary of the limitations of the evidence included in the review (e.g. study risk of bias, inconsistency and imprecision).	Yes
Interpretation	10	Provide a general interpretation of the results and important implications.	Yes
**OTHER**			
Funding	11	Specify the primary source of funding for the review.	Yes
Registration	12	Provide the register name and registration number.	Yes

From: Page MJ, McKenzie JE, Bossuyt PM, Boutron I, Hoffmann TC, Mulrow CD, et al. The PRISMA 2020 statement: an updated guideline for reporting systematic reviews. BMJ 2021;372:n71. doi: 10.1136/bmj.n71. This work is licensed under CC BY 4.0. To view a copy of this license, visit https://creativecommons.org/licenses/by/4.0/

PRISMA 2020 Checklist

Section and Topic	Item #	Checklist item	Location where item is reported
**TITLE**			
Title	1	Identify the report as a systematic review.	1
**ABSTRACT**			
Abstract	2	See the PRISMA 2020 for Abstracts checklist.	1
**INTRODUCTION**			
Rationale	3	Describe the rationale for the review in the context of existing knowledge.	3-4
Objectives	4	Provide an explicit statement of the objective(s) or question(s) the review addresses.	3-4
**METHODS**			
Eligibility criteria	5	Specify the inclusion and exclusion criteria for the review and how studies were grouped for the syntheses.	4
Information sources	6	Specify all databases, registers, websites, organisations, reference lists and other sources searched or consulted to identify studies. Specify the date when each source was last searched or consulted.	5
Search strategy	7	Present the full search strategies for all databases, registers and websites, including any filters and limits used.	OSF-project
Selection process	8	Specify the methods used to decide whether a study met the inclusion criteria of the review, including how many reviewers screened each record and each report retrieved, whether they worked independently, and if applicable, details of automation tools used in the process.	5
Data collection process	9	Specify the methods used to collect data from reports, including how many reviewers collected data from each report, whether they worked independently, any processes for obtaining or confirming data from study investigators, and if applicable, details of automation tools used in the process.	5
Data items	10a	List and define all outcomes for which data were sought. Specify whether all results that were compatible with each outcome domain in each study were sought (e.g. for all measures, time points, analyses), and if not, the methods used to decide which results to collect.	6
	10b	List and define all other variables for which data were sought (e.g. participant and intervention characteristics, funding sources). Describe any assumptions made about any missing or unclear information.	6, OSF
Study risk of bias assessment	11	Specify the methods used to assess risk of bias in the included studies, including details of the tool(s) used, how many reviewers assessed each study and whether they worked independently, and if applicable, details of automation tools used in the process.	6
Effect measures	12	Specify for each outcome the effect measure(s) (e.g. risk ratio, mean difference) used in the synthesis or presentation of results.	6-7
Synthesis methods	13a	Describe the processes used to decide which studies were eligible for each synthesis (e.g. tabulating the study intervention characteristics and comparing against the planned groups for each synthesis (item #5)).	6-7
	13b	Describe any methods required to prepare the data for presentation or synthesis, such as handling of missing summary statistics, or data conversions.	6-7
	13c	Describe any methods used to tabulate or visually display results of individual studies and syntheses.	6-7
	13d	Describe any methods used to synthesize results and provide a rationale for the choice(s). If meta-analysis was performed, describe the model(s), method(s) to identify the presence and extent of statistical heterogeneity, and software package(s) used.	6-7
	13e	Describe any methods used to explore possible causes of heterogeneity among study results (e.g. subgroup analysis, meta-regression).	na
	13f	Describe any sensitivity analyses conducted to assess robustness of the synthesized results.	6-7
Reporting bias assessment	14	Describe any methods used to assess risk of bias due to missing results in a synthesis (arising from reporting biases).	7
Certainty assessment	15	Describe any methods used to assess certainty (or confidence) in the body of evidence for an outcome.	7

PRISMA 2020 Checklist

Section and Topic	Item #	Checklist item	Location where item is reported
**RESULTS**			
Study selection	16a	Describe the results of the search and selection process, from the number of records identified in the search to the number of studies included in the review, ideally using a flow diagram.	8
	16b	Cite studies that might appear to meet the inclusion criteria, but which were excluded, and explain why they were excluded.	8
Study characteristics	17	Cite each included study and present its characteristics.	8-17
Risk of bias in studies	18	Present assessments of risk of bias for each included study.	18-20
Results of individual studies	19	For all outcomes, present, for each study: (a) summary statistics for each group (where appropriate) and (b) an effect estimate and its precision (e.g. confidence/credible interval), ideally using structured tables or plots.	9-13
Results of syntheses	20a	For each synthesis, briefly summarise the characteristics and risk of bias among contributing studies.	20-22
	20b	Present results of all statistical syntheses conducted. If meta-analysis was done, present for each the summary estimate and its precision (e.g. confidence/credible interval) and measures of statistical heterogeneity. If comparing groups, describe the direction of the effect.	na
	20c	Present results of all investigations of possible causes of heterogeneity among study results.	na
	20d	Present results of all sensitivity analyses conducted to assess the robustness of the synthesized results.	na
Reporting biases	21	Present assessments of risk of bias due to missing results (arising from reporting biases) for each synthesis assessed.	na
Certainty of evidence	22	Present assessments of certainty (or confidence) in the body of evidence for each outcome assessed.	20-22
**DISCUSSION**			
Discussion	23a	Provide a general interpretation of the results in the context of other evidence.	22-23
	23b	Discuss any limitations of the evidence included in the review.	23-24
	23c	Discuss any limitations of the review processes used.	24-25
	23d	Discuss implications of the results for practice, policy, and future research.	25
**OTHER INFORMATION**			
Registration and protocol	24a	Provide registration information for the review, including register name and registration number, or state that the review was not registered.	Cover page
	24b	Indicate where the review protocol can be accessed, or state that a protocol was not prepared.	Cover page
	24c	Describe and explain any amendments to information provided at registration or in the protocol.	Cover page
Support	25	Describe sources of financial or non-financial support for the review, and the role of the funders or sponsors in the review.	26
Competing interests	26	Declare any competing interests of review authors.	26
Availability of data, code and other materials	27	Report which of the following are publicly available and where they can be found: template data collection forms; data extracted from included studies; data used for all analyses; analytic code; any other materials used in the review.	OSF

From: Page MJ, McKenzie JE, Bossuyt PM, Boutron I, Hoffmann TC, Mulrow CD, et al. The PRISMA 2020 statement: an updated guideline for reporting systematic reviews. BMJ 2021;372:n71. doi: 10.1136/bmj.n71.
This work is licensed under CC BY 4.0. To view a copy of this license, visit https://creativecommons.org/licenses/by/4.0/

PRISMA-P 2015 Checklist

This checklist has been adapted for use with protocol submissions to *Systematic Reviews* from Table 3 in Moher D et al: Preferred reporting items for systematic review and meta-analysis protocols (PRISMA-P) 2015 statement. *Systematic Reviews* 2015 **4**:1

Section/topic	#	Checklist item	Information reported		Line number(s)
			Yes	No	
**ADMINISTRATIVE INFORMATION**					
**Title**					
Identification	1a	Identify the report as a protocol of a systematic review	☒	☐	Page 1
Update	1b	If the protocol is for an update of a previous systematic review, identify as such	☐	☐	NA
**Registration**	2	If registered, provide the name of the registry (e.g., PROSPERO) and registration number in the Abstract	☒	☐	Page 1
**Authors**					
Contact	3a	Provide name, institutional affiliation, and e-mail address of all protocol authors; provide physical mailing address of corresponding author	☒	☐	Page 1
Contributions	3b	Describe contributions of protocol authors and identify the guarantor of the review	☒	☐	Page 1
**Amendments**	4	If the protocol represents an amendment of a previously completed or published protocol, identify as such and list changes; otherwise, state plan for documenting important protocol amendments	☒	☐	Page 1, C13
**Support**					
Sources	5a	Indicate sources of financial or other support for the review	☒	☐	Page 1
Sponsor	5b	Provide name for the review funder and/or sponsor	☒	☐	Page 1
Role of sponsor/funder	5c	Describe roles of funder(s), sponsor(s), and/or institution(s), if any, in developing the protocol	☒	☐	Page 1
**INTRODUCTION**					
**Rationale**	6	Describe the rationale for the review in the context of what is already known	☒	☐	C1, C4
**Objectives**	7	Provide an explicit statement of the question(s) the review will address with reference to participants, interventions, comparators, and outcomes (PICO)	☒	☐	C1, C2

BioMed Central
The Open Access Publisher

Section/topic	#	Checklist item	Information reported Yes	Information reported No	Line number(s)
**METHODS**					
**Eligibility criteria**	8	Specify the study characteristics (e.g., PICO, study design, setting, time frame) and report characteristics (e.g., years considered, language, publication status) to be used as criteria for eligibility for the review	☒	☐	C5, C6, C7, C8, C9
**Information sources**	9	Describe all intended information sources (e.g., electronic databases, contact with study authors, trial registers, or other grey literature sources) with planned dates of coverage	☒	☐	C19
**Search strategy**	10	Present draft of search strategy to be used for at least one electronic database, including planned limits, such that it could be repeated	☒	☐	C19
STUDY RECORDS					
Data management	11a	Describe the mechanism(s) that will be used to manage records and data throughout the review	☒	☐	Page 1
Selection process	11b	State the process that will be used for selecting studies (e.g., two independent reviewers) through each phase of the review (i.e., screening, eligibility, and inclusion in meta-analysis)	☒	☐	C19
Data collection process	11c	Describe planned method of extracting data from reports (e.g., piloting forms, done independently, in duplicate), any processes for obtaining and confirming data from investigators	☒	☐	Page 1
**Data items**	12	List and define all variables for which data will be sought (e.g., PICO items, funding sources), any pre-planned data assumptions and simplifications	☒	☐	C14, C15
**Outcomes and prioritization**	13	List and define all outcomes for which data will be sought, including prioritization of main and additional outcomes, with rationale	☒	☐	C14, C15
**Risk of bias in individual studies**	14	Describe anticipated methods for assessing risk of bias of individual studies, including whether this will be done at the outcome or study level, or both; state how this information will be used in data synthesis	☒	☐	C20
DATA					
**Synthesis**	15a	Describe criteria under which study data will be quantitatively synthesized	☒	☐	C21
	15b	If data are appropriate for quantitative synthesis, describe planned summary measures, methods of handling data, and methods of combining data from studies, including any planned exploration of consistency (e.g., I^2, Kendall's tau)	☒	☐	C21
	15c	Describe any proposed additional analyses (e.g., sensitivity or subgroup analyses, meta-regression)	☒	☐	C22
	15d	If quantitative synthesis is not appropriate, describe the type of summary planned	☒	☐	C21
**Meta-bias(es)**	16	Specify any planned assessment of meta-bias(es) (e.g., publication bias across studies, selective	☒	☐	Page 2

Section/topic	#	Checklist item	Information reported		Line number(s)
			Yes	No	
		reporting within studies)			
**Confidence in cumulative evidence**	17	Describe how the strength of the body of evidence will be assessed (e.g., GRADE)	☒	☐	C23

BioMed Central
The Open Access Publisher

Cologne, 12th August 2025

A priori protocol following PRISMA-P (2015) and integrated by the standards for the conduct of new Cochrane Intervention Reviews (MECIR) https://www.cochrane.org/authors/handbooks-and-manuals/mecir-manual/standards-conduct-new-cochrane-intervention-reviews-c1-c75/developing-protocol-review-c1-c23
The two guidelines have been integrated since no new version of PRISMA-P was released in the 2020 guidelines, and the standard for the conduct outlined in the MECIR is more stringent.

PRISMA-P Checklist (2015):
1a: Acceptance and Commitment Therapy (ACT) in the pediatric psycho-oncology: a systematic review
1b: NA
2: This protocol is an a priori protocol that will be uploaded to the PROSPERO framework. A pre-registration code will be assigned to the review after the upload of this document.
3a/b: See „Review team members", „Review affiliation" and „Funding source" in the PROSPERO registration.
4: Further amendments will be recorded in the PROSPERO framework (also see C13)
5a/b/c: The systematic review was funded by employment at the University of Cologne. The institution had no role in design, data collection, analysis, decision to publish, or manuscript preparation. All authors declare no known competing financial or personal interests.
6: See C1 and C4.
7: See C1 and C2.
8: See C5, C6, C7, C8 and C9.
9: See C19.
10: See C19.
11a: Endnote 21 will be used to manage the records throughout the review.
11b: See C19.
11c: One reviewer will extract data from each included study using a pre-piloted Excel extraction form (tested on two studies before full extraction). A second reviewer (or machine) will check the extracted data for accuracy and completeness. Data items will include study characteristics, participant details, intervention characteristics, comparator details, and reported outcomes. If data are missing or unclear, study authors will be contacted for clarification. Any disagreements during the data extraction process will be resolved by discussion or, if necessary, by consulting a third reviewer.
12: See C14 and C15.
13: See C14 and C15.
14: See C20.
15a: See C21.
15b: See C21.

15c: See C22.
15d: See C21.
16: If ≥10 studies in a specific sub-group are available for a given outcome, we will assess potential publication bias by visually inspecting funnel plots and, where appropriate, conducting statistical tests for asymmetry (e.g., Egger's test). Selective outcome reporting will be evaluated by comparing the outcomes reported in study protocols or trial registrations (where available) against those reported in the published results. Any discrepancies will be documented and discussed.
17: see C23.

Methodological Expectations of Cochrane Intervention Reviews (MECIR)

Research questions

C1. Formulating review questions

The aim of this systematic review is to identify and synthesize published evidence on the use of interventions based on Acceptance and Commitment Therapy (ACT) in pediatric psycho-oncology. Specifically, the review will address whether and how ACT has been applied in the following groups:

- Children, adolescents and young adults (<25 years) undergoing cancer treatment or who have survived childhood cancer;
- Children, adolescents and young adults (<25 years) whose parent(s) has cancer (parental cancer);
- Parents of children, adolescents, and young adults (<25 years) with cancer and who are undergoing treatment OR who have survived childhood cancer.

The review will focus on evaluating the impact of these interventions on quantitative outcomes such as fear of cancer recurrence (FCR), quality of life (QoL), psychological flexibility, ACT-specific measures (e.g., cognitive defusion, acceptance …), symptoms of mental disorders (e.g., depression, anxiety, PTBS, …) and other related psychological measures.

This question is highly relevant for health professionals, researchers, and policy makers seeking evidence-based psychosocial interventions in pediatric oncology. Given the substantial functional and emotional challenges faced by children, adolescents, young adults and families affected by cancer, understanding the effectiveness and implementation of ACT in this context is crucial. While ACT has shown promising results in adult psycho-oncology settings (e.g., Arch & Mitchell, 2016; Feros et al., 2013; Jiang et al., 2024), its use in pediatric populations remains underexplored. This review will help inform the development and adaptation of evidence-based psychosocial interventions for children, adolescents, young adults and families, addressing a critical gap in the research of pediatric psycho-oncology.

C2. Predefining objectives

The PICOS criteria for this study are:

P (Population): patients under 25 years old (childhood and AYAs) who are currently receiving cancer treatment or are pediatric cancer survivors OR whose parents have cancer (parental cancer), OR/AND parents whose children (<25) have cancer or are childhood cancer survivors;
I (Intervention): ACT, ACT-based interventions, and further adaptations of ACT;
C (Control): any or none (including usual care, active control, or no control);
O (Outcomes): any reported psychosocial, behavioral, or health-related outcomes assessed through psychometric instruments;
S (Study design): quantitative (e.g., RCTs, non-randomized trials, pre-post studies, single-case designs).
Since there are three subgroups, the interventions will be summarized separately by subgroup.

C3. Considering potential adverse effects
Although ACT is generally considered a safe, non-invasive psychological intervention, this review will take into account any reported adverse effects or unintended negative outcomes associated with ACT-based interventions in the pediatric psycho-oncology context. Potential adverse effects could include increased emotional distress, resistance to treatment, worsening of symptoms, or negative family dynamics triggered by therapy content and by the promotion of acceptance and mindful presence with cancer related images, memories and emotions.

C4. Considering equity and specific populations
Cancer is a major predisposing factor that significantly increases the risk of psychological distress and mental health disorders in children and adolescents (AWMF, 2023, p. 43). These consequences stem not only from the long-term physical side effects of treatment (e.g., chemotherapy, radiotherapy) and the disease itself (Eiser, 1998), but also from the disruption or delay of age-appropriate developmental tasks (Schröder et al., 2019, p. 8), fear of cancer recurrence (FCR), intrusive memories, social isolation, and increased rates of school dropout due to prolonged absences (Tremolada et al., 2022; Signorelli et al., 2017).
Psychosocial interventions in pediatric psycho-oncology typically extend beyond the child patient to include the broader family system, which is often significantly affected by the diagnosis (Neugebauer & Mastergeorge, 2021; Schröder et al., 2019). For primary caregivers, childhood cancer can result in elevated stress levels, anxiety, depression, and post-traumatic stress symptoms (Sultan et al., 2016), impaired quality of life (Klassen et al., 2011), and financial hardship due to caregiving demands and lost work time (Neugebauer & Mastergeorge, 2021).
These populations are inherently vulnerable, both medically and psychosocially. Where data are available, this review will explore how ACT-based interventions are applied across diverse subgroups, including those from lower socioeconomic backgrounds, underrepresented ethnic or cultural groups, and families in low- and middle-income countries (LMICs). We will examine whether interventions were culturally, developmentally, or linguistically adapted, and whether any disparities in access, engagement, or outcomes were reported.

Given ACT's emphasis on assessing patients' sense of meaning through work on values, consideration of the patient's religious context is essential for ensuring the acceptability of the therapeutic intervention. We will consider whether the studies have assessed the religiosity and spirituality of patients.

Equity-related findings, such as differential intervention effects by socioeconomic status, gender, ethnicity, or region, will be extracted and summarized narratively, where possible. This will support a broader understanding of the potential for ACT-based interventions not only to promote psychosocial well-being, but also to reduce disparities in pediatric psycho-oncology care.

Setting the eligibility criteria for including studies in the review

C5. Predefining unambiguous criteria for participants

The review will include studies involving the following participant groups:

- Children, adolescents and young adults under 25 years of age who are either:
  - Diagnosed with cancer and currently undergoing treatment, or
  - Pediatric cancer survivors (any time post-treatment).
- Children, adolescents and young adults (<25 years), whose parents have cancer and are currently undergoing treatment;
- Parents or primary caregivers of children, adolescents and young adults under 25 years of age who have been diagnosed with cancer or are childhood cancer survivors.

These populations were chosen because childhood cancer affects not only the patient but also the immediate family system, with documented psychological consequences for both children, adolescents and young adults and their parents or caregivers.

All types and forms of malignant cancer diagnoses (i.e., cancers with potential for progression, metastasis, or recurrence) will be included (e.g., hematologic malignancies, solid tumors, central nervous system cancers), with no restriction based on cancer type, stage, or treatment modality. This inclusive approach is intended to capture the full range of psycho-oncological experiences in pediatric populations.

C6. Predefining a strategy for studies with a subset of eligible participants

Given the inconsistent definition of young adults (individuals between the ages 18 and 25 or 18 and 39), criterion C6 holds significant importance. For synthesis, we will extract subgroup-specific data wherever reported.

If subgroup data remain unavailable, the study will be included in the review and risk-of-bias assessment, but excluded from the formal subgroup synthesis.

Exception: if ≥80% of participants fall within a predefined age band, or the reported age range lies entirely within that band, we will include the combined data in that subgroup's synthesis and note the approximation.

C7. Predefining unambiguous criteria for interventions and comparators

Eligible interventions will include Acceptance and Commitment Therapy (ACT) and ACT-based adaptations. This includes interventions explicitly grounded in ACT principles (e.g., psychological flexibility, values-based action, mindfulness) and recognized models derived from ACT, such as the DNA-V model (Hayes & Ciarrochi, 2015). Variations in setting (e.g., hospital, outpatient, online), delivery format (e.g., individual, group, digital), duration, intensity, and provider (e.g., psychologist, counselor, trained facilitator) will be allowed.

Studies in which ACT techniques are embedded only minimally within broader cognitive-behavioral therapy (CBT) protocols, or where ACT is not the primary therapeutic orientation, will be excluded.

All types of comparators (e.g, inactive controls, active controls and no comparator) will be considered. No restrictions will be applied regarding comparator intensity, duration, or delivery mode.

C8. Clarifying role of outcomes

The review will not use outcome measures as inclusion criteria. Studies will be included based on population and intervention criteria, regardless of which outcomes they report. Outcomes such as fear of cancer recurrence (FCR), quality of life (QoL), psychological flexibility, and other ACT-specific or health-related measures are of primary (but not exclusive) interest.

C9. Predefining study designs

Quantitative studies of any design will be considered eligible, provided they include at least one quantifiable psychosocial, behavioral, or health-related outcome assessed before and/or after the intervention. Eligible designs include, but are not limited to:

- Randomized controlled trials (RCTs);
- Non-randomized controlled trials;
- Quasi-experimental studies;
- Uncontrolled pre-post studies;
- Single-group longitudinal studies;
- Single-case designs with repeated measurement (e.g., AB, ABA designs).

No restrictions will be placed on sample size, blinding, allocation procedures, or the presence or type of a comparator. Studies will not be excluded based on risk of bias at the eligibility stage. Qualitative studies will be excluded from the primary review.

C10. Including randomized trials

Randomized controlled trials (RCTs) will be eligible for inclusion in this review. No restrictions will be applied based on randomization procedures, blinding, or follow-up duration. All RCTs that meet the population and intervention criteria will be assessed for inclusion.

C11. Justifying choice of study designs

This review will include a broad range of quantitative study designs (e.g., non-randomized trials, pre-post studies, single-case designs, and other observational designs with quantifiable outcomes). This liberal inclusion criterion is justified by the emerging nature of ACT research in pediatric oncology settings. Including diverse study designs will allow for a more comprehensive understanding of how ACT is being used in practice, and what preliminary evidence exists regarding its effectiveness.

C12. Excluding studies based on publication status

In addition to peer-reviewed journal articles, grey literature sources, such as preprints, dissertations, theses, and research reports will be included in the review, provided that sufficient methodological detail (description of the intervention and reporting of outcomes) is available and that the full text is accessible.

By incorporating grey literature, a comprehensive global perspective on the research on the application of ACT in pediatric psycho-oncology will be presented. Achieving this objective will be challenging, as the identification of grey literature is more difficult compared to peer-reviewed published studies. These limitations will be further elaborated upon.

When multiple publications report on the same study, we will collate them and treat them as a single study, prioritizing the most complete dataset. Non-experimental studies that are commentaries or reviews of other studies will be formally excluded but may be discussed in the discussion section. Errata of eligible studies are going to be considered in the inclusion/exclusion process.

C13. Changing eligibility criteria

We do not anticipate making changes to the pre-specified eligibility criteria. In the event of unforeseen challenges or findings during the review process, such as ambiguous reporting in primary studies, unexpected variations in study design, or a substantial proportion of qualitative study designs, any modifications to eligibility criteria will be implemented in a transparent and justified manner.

Selecting outcomes to be addressed for studies included in the review

C14. Predefining outcome domains

The following outcomes have been defined in advance for this review. All outcomes must be assessed using psychometric instruments.

- Psychological flexibility, measured through instruments such as the Acceptance and Action Questionnaire (AAQ-II) or Avoidance and Fusion Questionnaire for Youth (AFQ-Y) or Parent Psychological Flexibility Questionnaire (PPFQ), Children's Psychological Flexibility Questionnaire (CPFQ);
- Fear of cancer recurrence (FCR), measured through validated instruments (e.g., FCRI).
- Quality of life, using measures such as EuroQol 5-dimensions youth-version, WHO Well-Being Index 5-items;

- Symptoms of other mental disorders (e.g., anxiety, depression or PTSD), measured using instruments such as the CBCL, YSR, SDQ, and others.
- Mindfulness, measured by instruments such as the CAMM (Child and Adolescent Mindfulness Measure) or MAAS and MAAS-A
- Acceptance and experiential avoidance, assessed through tools like the Parental Acceptance Questionnaire (6-PAQ), Parental Acceptance and Action Questionnaire (PAAQ), Children's Psychological Flexibility Questionnaire (CPFQ);
- Cognitive fusion, evaluated using the Cognitive Fusion Questionnaire (CFQ-7)
- Feasibility of the intervention, including measures of recruitment, retention, adherence, and completion rates reported in studies.
- Acceptability of the intervention, including participant satisfaction, qualitative feedback, and dropout reasons related to the intervention.

Potential areas of harm include the exacerbation of PTSD and anxiety-related symptoms by promoting acceptance and mindful presence in the presence of cancer-related images, memories, and emotions.

C15. Choosing outcomes

The outcomes selected in this review were chosen based on their relevance to patients and their families, mental health professionals, and policymakers working in pediatric psycho-oncology. The review will prioritize outcomes that are recorded using patient-reported instruments.

The following outcomes were selected:

Critical outcomes (GRADE-outcomes)

- Psychological (in)flexibility (as the core mechanism of ACT);
- Fear of cancer recurrence (FCR);
- Quality of life (QoL).
- Symptoms of any other mental disorder (such as screening scores of depression, PTSD and/or anxiety);
- Acceptability of the intervention (e.g., participant satisfaction, perceived usefulness, cultural adaptation):
- Feasibility of the intervention (e.g., recruitment, retention, adherence rates);
- Health-related resource use or cost (e.g., if group or individual or if delivered by psychotherapists or other mental health professionals, and costs related to training for the intervention).

Additional outcomes include:

- Other ACT-specific outcomes (e.g., Acceptance, Self-as-Context, Mindfulness)

These last outcomes are considered additional because they are interim outcomes of psychological flexibility in the ACT framework.

Planning the review methods at protocol stage

C19. Planning the search

The following search term was developed using PICOS criteria and eligibility criteria:

Table 1.

**Search strategy**	(child* OR adolescent* OR youth OR teen* OR bambin* OR parent* OR Eltern OR genitor* OR mother* OR father* OR madr* OR padr*)<br>AND („Acceptance and Commitment Therapy“ OR „Acceptance- and Commitment-Therapy“ OR „Akzeptanz- und Commitment-Therapie“ OR „ACT-based“)<br>AND (cancer OR neoplasm* OR Krebs OR Tumor* OR neoplasi* OR neoplastic diseases OR psychooncology OR psycho-oncology OR Psychoonkologie OR Psycho-onkologie OR psico-oncologia OR psicooncologia)

The search term encompasses terms in English, Italian, and German (reflecting team language competencies), thereby enabling a comprehensive search of articles, thus minimizing language bias. Non-English/Italian/German full texts will be translated, where necessary, using machine translation (no language restriction). The term will undergo modification to conform to the preferred formats of the following databases. For example for PubMed:

Table 2.

**Search strategy**	("Child"[Mesh] OR "Adolescent"[Mesh] OR "Parents"[Mesh] OR child*[tiab] OR adolescent*[tiab] OR youth[tiab] OR teen*[tiab] OR bambin*[tiab] OR parent*[tiab] OR Eltern[tiab] OR genitor*[tiab] OR mother*[tiab] OR father*[tiab] OR madr*[tiab] OR padr*[tiab])<br>AND ("Acceptance and Commitment Therapy"[Mesh] OR "Acceptance and Commitment Therapy"[tiab] OR "Acceptance- and Commitment-Therapy"[tiab] OR "Akzeptanz- und Commitment-Therapie"[tiab] OR ACT-based[tiab])<br>AND ("Neoplasms"[Mesh] OR "Psycho-Oncology"[Mesh] OR cancer[tiab] OR neoplasm*[tiab] OR Krebs[tiab] OR Tumor*[tiab] OR Neoplasie[tiab] OR "neoplastic diseases"[tiab] OR psychooncology[tiab] OR "psycho-oncology"[tiab] OR Psychoonkologie[tiab] OR "Psycho-onkologie"[tiab] OR "psico-oncologia"[tiab] OR psicooncologia[tiab])
Databases: PubMed	

The selection of the databases was made in accordance with the guidelines outlined in the manual „Doing a Systematic Review: A Student's Guide“ (Boland et al., 2017) and integrated trough the recommendations of the AI program Search Smart (https://www.searchsmart.org/).

Table 3.

**Database**	**Platfrom**	**Coverage (approx.)**

MEDLINE	EBSCOhost	1879 - present
APA PsycInfo	EBSCOhost	1967 - present
PsyIndex	EBSCOhost	1977 - present
LIVIVO	ZB MED-Suchportal	2015 - present
Web of Science Core Collection	Clarivate	1945 - present
Research Gate	https://www.researchgate.net/search	2008 - present
EMBASE	ZB MED-Suchportal	1947 - present
CINAHL	EBSCOhost	1937 - present
Europe PMC	https://europepmc.org/	1768 - present
WHO International Clinical Trials Registry Platform	https://trialsearch.who.int/	1990 - present
PubMed	https://pubmed.ncbi.nlm.nih.gov/	1879 - present
DANS Data Station Life Sciences	https://lifesciences.datastations.nl/dataverse/root	2005 - present
DANS Data Station Social Sciences and Humanities	https://ssh.datastations.nl/	1957 - present
EThOS	https://bl.iro.bl.uk/concern/datasets/10cc13f9-797d-41f2-a7e2-d29f4306133e	1917 - present
ClinicalTrials.gov	clinicaltrials.gov	1947 - present

The use of the databases ResearchGate, WHO ICTRP, DANS, and EThOS serves to reduce the risk of publication bias, as they include some or only grey literature.

No date or publication status restrictions will be applied.

Reference lists of included studies and relevant systematic reviews will be manually screened to identify additional eligible studies. We will use AI-tools (e.g., Research Rabbit) to facilitate backward and forward citation.

All search terms and the date of the last database search will be included in the appendix.

The studies will be screened by one person (or a machine) and checked by at least one other person (or machine). Title and abstract will be screened, then full text. Screening and de-duplication will be managed using EndNote 21.

C20. Planning the assessment of risk of bias in included studies

Given the anticipated heterogeneity and limited number of studies, we will carefully document the risk of bias for each study.

For randomized controlled trials, the Cochrane Risk of Bias 2 (RoB 2) tool will be used. For non-randomized studies, ROBINS-I-V2 (Risk Of Bias In Non-randomized Studies - of Interventions - Version 2) will be used.

Results of risk of bias assessments will be presented in a summary figure (for RoB 2) and a table (for ROBINS-I-V2), as recommended by the Cochrane Handbook.
The risk of bias of the studies will be assessed by one person (or a machine). A random 15% of the studies will undergo peer review by at least one other researcher or automated system. Disagreements will be resolved by discussion and by contacting a third reviewer if necessary.
The quality of this systematic review will be assessed through the checklist of the PRISMA-2020 Guidelines.

C21. Planning the synthesis of results.
Given the anticipated small number of studies and expected heterogeneity in populations, interventions, and outcomes, a meta-analysis will not be conducted. Instead, findings will be synthesized narratively in accordance with the Synthesis Without Meta-analysis (SWiM) reporting guidelines (Campbell et al., 2019) and Cochrane Handbook Chapter 12.
Primary grouping will be by population (see PICOS). Within each population, outcomes will be synthesized by domain and timepoint (post-treatment; follow-ups). Within each grouping, results will be summarized by direction of effect and magnitude where possible (e.g., improvement, no change, deterioration), along with the effect size metrics reported by the original studies (e.g., mean difference, standardized mean difference). Any patterns in study design, quality/risk of bias, and contextual factors will be described alongside the results to aid interpretation.

C22. Planning sub-group analyses.
Given the anticipated heterogeneity and small number of studies, we do not plan a pooled meta-analysis. Where appropriate and if sufficient homogenous data become available, exploratory subgroup meta-analyses may be considered; otherwise subgroup comparisons will be performed narratively.

C23. Planning the GRADE assessment and ‘Summary of findings’ table.
The certainty of evidence for predefined critical outcomes will be assessed using GRADE. The Summary of Findings (SoF) table will include ≤7 critical outcomes (C15), prioritized by importance to patients, families, and clinicians in pediatric psycho-oncology.
One reviewer will conduct the GRADE assessments. A random 20% of GRADE assessments will undergo peer review by at least one other researcher or automated system.
Summary of Findings (SoF) tables will be generated in GRADEpro.
Additional ACT-specific outcomes are not going to be included in the GRADE table since they are intended as interim outcomes of psychological flexibility.
