# Supplementary material for: Acceptance and Commitment Therapy for Children, Adolescents, and Young Adults With Cancer and Their Parents and for Children, Adolescents, and Young Adults Affected by Parental Cancer: A Population‐Stratified Systematic Review
Source: Brain Behav. 2026 Nov 9;16(11):e71902. doi: 10.1002/brb3.71902 (PMC13746990; doi:10.1002/brb3.71902)
Supplement: Supplementary file 2 [file BRB3-16-e71902-s001.docx]

**Supplemental Table S1**
*Extraction table of included studies.*

| **Author, Year and Country/Region** | **Study Design** | **Participants characteristics (N, mean age, SD and range, diagnosis condition, time since diagnosis)** | **Description of the intervention (session number, duration of a session, frequency. delivery mode, provider qualifications, control group)** | **Time points of data collection (except screening)** | **Quantitative outcomes and measurments tools (except screening)** | **Results (only significant: *p* < 0.05)** | **(Session) attendence, *N* at each timepoint** |  |
| --- | --- | --- | --- | --- | --- | --- | --- | --- |
| **Clarke et al. (2021)**  **Australia** | Single group process evaluation | ***N*** = 28 Adolescents who had been diagnosed with cancer, and who had completed curative can-cer treatment or were receiving long-term or maintenance therapy  **Age**: *M* (*SD*) = 15.4 (1.6)  **Condition**: Cancer (96%), 4 % similar treatment (chemotherapy) without cancer diagnosis  **Time since diagnosis**: Any (53% adolescence) | **Dose**: 90 minutes, 5 sessions over three days. **Delivery mode**: Camp-based intervention. **Provider qualification**: Psychosocial workers with general ACT training. | End of each session | 1) Program acceptability (author-made satisfaction questionnaires). 2) Program Feasibility (author-made implementation fidelity questionnaires only for the facilitators). | Only process evaluation.  Acceptability: 85% rated sessions as helpful, 89% as meaningful. 2) Implementation fidelity: 70% activities delivered as per manual; 39% of facilitators self-reporting very/extremely competent delivery. | Attendence: 97%. *N* = 28 at each timepoint. |  |
| **Patterson et al. (2022)**  **Australia** | Pre-Post Study With No Control Group | ***N*** = 148 (full evaluation cohort; analytic subsample n = 52) Distressed adolescents impacted by their own or parental cancer  **Age**: *M* (*SD*) = 14.5 (1.6)  **Condition**: Cancer or family member’s cancer  **Time since diagnosis**: Any type or stage of cancer in the previous 5 years | **Dose**: 90 minutes, four days. **Delivery mode**: Group, camp based. **Provider qualification**: Psycho-social workers with ACT training. | T0: Baseline  T1: Immediately post-program (QoL only)  T2: 2 months after T1 | 1) QoL (author-made questionnaire). 2) Psychological distress (K10). 3) Peer support (CaPSS). 4) Psychological inflexibility (AFQ-Y8). 5) Self-compassion (SCS). 6) Mindfulness (CAMM). | 1) Quality of Life: 20.3% experienced clinically meaningful improvement T0→T1 (*d* ≥ 0.5). 15.0% experienced clinically meaningful improvement T0→T2 (*d* ≥ 0.5) 2) Psychological Distress: 18.0% experienced clinically meaningful improvement T0→T2 (*d* ≥ 0.5). No significant mean-level change reported. *M*/*SDs* not reported; effect sizes cannot be calculated. Psychological distress significantly mediated QoL improvement (*β* = 0.77, *SE* = 0.20, 95% CI [0.39, 1.19]). 4) Psychological Inflexibility: data collected but detailed results not reported. Psychological flexibility significantly mediated QoL improvement (*β* = 0.21, *SE* = 0.11, 95% CI [0.002, 0.442]). *M*/*SDs*: Not reported; effect sizes cannot be calculated. 5) Self-Compassion: data collected but detailed results not reported Self-compassion (self-kindness) significantly mediated QOL improvement (*β* = 0.26, *SE* = 0.12, 95% CI [0.04, 0.51]). *M*/*SDs* not reported; effect sizes cannot be calculated. ^†^ | Attendence: not stated. Assessed for eligibility: *N* = 158. Inclusion criteria met: *N* = 148. T0: *N* = 148; T1: *N* = 148; T2: *N* = 102. | Patterson et al. (2021): Process evaluation. Patterson et al. (2015) Study protocol. |
| **Stuart (2022)**  **USA** | Pre-Post Study With No Control Group | ***N*** = 11 Adolescents and Young Adults who suvrived Childhood Cancer^‡^  **Age**: *M* (*SD*) = 21.4 (2.17)  **Condition**: Cancer  **Time since diagnosis**: Any | **Dose**: 90 minutes, weekly. **Delivery mode**: Group, online. **Provider qualification**: not reported. | T0: Before the intervention  T1: After the 5th session | 1) Depression (PHQ-9), 2) Generalized Anxiety (GAD-7), 3) Post-traumatic stress (PCL-5), 4) Interpersonal skills (IF), 5) Neurocognitive Functioning (NSSR), 6) Cognitive Flexibility (CFS). 7) Healthpromoting behaviors (HB). | No significant outcome. | Attendence: 100%. Assessed for eligibility: *N* = 445. Inclusion criteria met: *N* = 205. T0: *N* = 11. T1: *N* = 9. |  |
| **Bibby et al. (2024)**  **Australia** | Pragmatic controlled trial (non-random allocation) | ***N*** = 142 AYAs with a parent who was receiving treatment for cancer or had been diagnosed with cancer (EG: *N* = 77, CG(WL): *N* = 65)  **Age**: EG: *M* (*SD*) = 16.84 (2.82), CG: *M* (*SD*) = 17.11 (2.45)  **Condition**: Parental cancer  **Time since diagnosis**: In the last 5 years | **Dose**: 120 minutes, weekly. **Delivery mode**: Group, in-person (17 groups) or online (4 groups). **Provider qualification**: Psycho-social workers with ACT training. | T0: Baseline  T1: After intervention  T2: Two months after T1 | 1) Unmet needs (OCNI). 2) Psychological distress (K10). 3) Psychological inflexibility (AFQ-Y8). 4) Mindfulness (CAMM). 5) Family functioning (FRI). | 1) Unmet needs ↓ (Intervention: T0 *M* (*SD*) = 103.66 (30.70), T2 *M* (*SD*): not reported (estimated *M* = 87.9). Control: T0 *M* (*SD*) = 100.85 (35.11), T2 *M* (*SD*): not reported (estimated *M* = 94.6); between-group: *d* = 0.29 [-0.04, 0.62])§. | Attendence: 82.9%. Assessed for eligibility: *N* = 158. Inclusion criteria met: *N* = 147. EG: T0: *N* = 77; T1: *N* = 55; T2: *N* = 48. CG: T0: *N* = 65; T1: *N* = 47; T2: *N* = 37. ¶ | Allison et al. (2023) Acceptability and feasibility trial. Patterson et al. (2015) study protocol. Patterson et al. (2015) Process evaluation. |
| **Akbari et al. (2020)**  **Iran** | Quasi-experimental with control group | ***N*** = 30 mothers of children with cancer (EG: *N* = 15, CG(WL): *N* = 15)  **Age**: EG: *M* (*SD*) = 37.27 (2.86) CG: *M* *SD*) = 36.60 (2.87)  **Condition**: Child’s cancer  **Time of diagnosis**: Any | **Dose**: 120 minutes, weekly. **Delivery mode**: Group, in-person. **Provider qualification**: Clinical psychologists trained in ACT. | T0: Baseline  T1: After intervention completion | 1) Parental Self-Efficacy (PSAM) | 1) Parental Self-Efficacy ↑ (Intervention: T0 *M* (*SD*) = 31.40 (6.40), T1 *M* (*SD*) = 53.87 (13.35); Control: T0 *M* (*SD*) = 30.07 (5.73), T1 *M* (*SD*) = 29.00 (7.58); between-group *d* = 2.29 [1.33, 3.25]). | Attendence: 100%. Assessed for eligibility: *N* = not reported. Inclusion criteria met: *N* = 60. EG: T0: *N* = 15; T1: *N* = 15. CG: T0: *N* = 15; T1: *N* = 15. |  |
| **Baratian et al. (2017)**  **Iran** | Quasi-experimental with control group | ***N*** = 30 mothers of children with cancer (EG: *N* = 15, CG(NT): *N* = 15)  **Age**: EG: *M* (*SD*) = 30.45 (3.20), CG: *M* (*SD*) = 29.55 (2.91)  **Condition**: Child’s cancer  **Time of diagnosis**: Any | **Dose**: 120 minutes, weekly. **Delivery mode**: Group, in-person. **Provider qualification**: Not reported. | T0: Baseline  T1: After intervention completion | 1) Quality of Life (WHO-QOL-26) | 1) Quality of Life ↑ (Intervention: T0 *M* (*SD*) = 70.40 (6.40), T1 *M* (*SD*) = 92.97 (13.35); Control: T0 *M* (*SD*) = 69.07 (5.73), T1 *M* (*SD*) = 68.00 (7.58); between group *d* = 2.29 [1.37, 3.21]). | Attendence: 100%. Assessed for eligibility: *N* = 52. Inclusion criteria met: *N* = 30. EG: T0: *N* = 15; T1: *N* = 15. CG: T0: *N* = 15; T1: *N* = 15.. |  |
| **Bautista et al. (2023)**  **Spain** | Randomized non-concurrent multiple-baseline SCED | ***N*** = 12 Parents of a child in active treatment for cancer with medium or high psychosocial risk (measured with PAT)  **Age**: *M* (*SD*) = 41.42 (8.71)  **Condition**: Child’s cancer (most frequent leukemia)  **Time since diagnosis**: At least one month from diagnosis | **Dose**: 60 minutes, weekly. **Delivery mode**: Individual, online. **Provider qualification**: 2-year postgraduate training in ACT. | Aggregated T0: Repeated measurements before intervention (2- or 4-week baselines, daily ecological measures).  Aggregated T1: Measurements during the active intervention.  T2: 8-week follow-up after completing the second session. | 1) Depression and Anxiety (PHQ-4). 2) Repetitive negative thinking (RNTQ-3). 3) Valued living (VQ-3) | 1) Depression and Anxiety ↓ (T0 *M* (*SD*) = 5.07 (3.26), T2 *M* (*SD*) = 2.60 (1.81); *d* = 0.83 [0.26, 1.40]).  2) Repetitive Negative Thinking ↓ (T0 *M* (*SD*) and T2 *M* (*SD*) not stated; *d* = 0.81 [0.34, 1.28]).  3) Valued Living ↑ (T0 *M* (*SD*) and T2 *M* (*SD*) not stated; *d* = 1.07 [0.22, 1.91]). | Attendence: 100%. Assessed for eligibility: *N* = 27. Inclusion criteria met: *N* = 16. Discontinued during baseline: *N* = 3. Aggregated T0: *N* = 12. Aggregated T1: *N* = 12. T2: *N* = 12. |  |
| **Jin et al. (2025)**  **China** | RCT | ***N*** = 142 parents of children with cancer (EG: *N* = 71; CG(TAU): *N* = 71)  **Age**: EG: *M* (*SD*) = 34.99 (5.00) TAU: *M* (*SD*) = 35.17 (5.92)  **Condition**: Child’s cancer (91% hematological)  **Time since diagnosis**: Within 6 months | **Dose**: 45-60 minutes, weekly. **Delivery mode**: Individual, first session in person, sessions 2-4 with synchronous videos. **Provider qualification**: 14 full days ACT skill training and videotaped sessions reviewed by an ACT expert. | T0: baseline  T1: one week after intervention completion  T2: 3 months after T1 | 1) Parental psychological distress (DASS-21). 2) Quality of Life (PedsQL Parent HRQL). 3) Parental experience of child’s illness (PECI). 4) Experiential avoidance (AAQ-II). 5) Psychological flexibility (CompACT). 6) Child’s psychological adjustment (SDQ). | 1) Parent psychological distress ↓ (Intervention: T0 *M* (*SD*) = 35.18 (24.31), T2 *M* (*SD*) = 17.72 (17.33); Control: T0 *M* (*SD*) = 28.68 (20.89), T2 *M* (*SD*) = 22.50 (16.82); between-group *d* = 0.61 [0.27, 0.94]).  2) QoL ↑ (Intervention: T0 *M* (*SD*) = 56.06 (18.27), T2 *M* (*SD*) = 69.23 (17.83); Control: T0 *M* (*SD*) = 58.06 (16.36), T2 *M* (*SD*) = 63.39 (13.86); between-group *d* = 0.49 [0.16, 0.83]).  3) Guilt and worry ↓ (Intervention: T0 *M* (*SD*) = 2.33 (0.65), T2 *M* (*SD*) = 1.36 (0.66); Control: T0 *M* (*SD*) = 2.20 (0.71), T2 *M* (*SD*) = 1.97 (0.72); between-group *d* = 1.29 [0.93, 1.65]). Unresolved sorrow and anger ↓ (Intervention: T0 *M* (*SD*) = 2.18 (0.79), T2 *M* (*SD*) = 1.39 (0.77); Control: T0 *M* (*SD*) = 2.12 (0.76), T2 *M* (*SD*) = 1.88 (0.70); between-group *d* = 0.71 [0.37, 1.05]); Long-term uncertainty ↓ (Intervention: T0 *M* (*SD*) = 2.01 (0.98), T2 *M* (*SD*) = 1.20 (0.86); Control: T0 *M* (*SD*) = 2.02 (0.91), T2 *M* (*SD*) = 1.61 (0.80); between-group *d* = 0.41 [0.08, 0.75]);  Emotional resources ↑ (Intervention: T0 *M* (*SD*) = 2.20 (0.87), T2 *M* (*SD*) = 2.57 (0.94); Control: T0 *M* (*SD*) = 2.37 (0.80), T2 *M* (*SD*) = 2.36 (0.79); between-group *d* = 0.53 [0.19, 0.86]).  4) Experiential Avoidance ↓ (Intervention: T0 *M* (*SD*) = 25.49 (9.95), T2 *M* (*SD*) = 18.40 (9.03); Control: T0 *M* (*SD*) = 23.72 (9.43), T2 *M* (*SD*) = 22.18 (8.11); between-group *d* = 0.57 [0.24, 0.91]).  5) Psychological Flexibility ↑ (Intervention: T0 *M* (*SD*) = 61.34 (12.93), T2 *M* (*SD*) = 69.83 (11.82); Control: T0 *M* (*SD*) = 63.65 (12.07), T2 *M* (*SD*) = 65.23 (11.40); between-group *d* = 0.63 [0.30, 0.97]).  6) Child’s psychological difficulties ↓ (Intervention: T0 *M* (*SD*) = 13.56 (5.34), T2 *M* (*SD*) = 10.34 (4.91); Control: T0 *M* (*SD*) = 13.04 (5.12), T2 *M* (*SD*) = 11.93 (5.02); between-group *d* = 0.39 [0.06, 0.73]). | Attendence: 80%. Assessed for eligibility: *N* = 250. Inclusion criteria met: *N* = not stated. EG: T0: *N* = 71; T1: *N* = 70; T2: *N* = 65. CG: T0: *N* = 71; T1: *N* = 62; T2: *N* = 56. | Jin et al. (2023) Pilot randomised control trial. Jin et al. (2021) Clinical trial entry. Jin et al. (2021) RCT protocol. |
| **Kalateh et al. (2024)**  **Iran** | Quasi-experimental with control group | ***N*** = 30 mothers of children with cancer (EG: *N* = 15, CG(NT): *N* = 15)  **Age**: only range reported (25-45)  **Condition**: Child’s cancer  **Time of diagnosis**: Any | **Dose**: Not reported, weekly. **Delivery mode**: Group, in-person. **Provider qualification**: Not reported. | T0: Baseline  T1: After intervention completion.T2: Follow-up (timing not reported) | 1) Identity crisis (ICQ). 2) Distress tolerance (DTS) | 1) Identity crisis ↓ (Intervention: T0 *M* (*SD*) = 166.60 (30.09), T1 *M* (*SD*) = 114.43 (25.73); Control: T0 *M* (*SD*) = 158.02 (28.21), T1 *M* (*SD*) = 156.84 (27.42); between-group *d* = 1.60 [0.77, 2.42]; *η²* = 0.66). †† 2) Distress tolerance ↓ (Intervention: T0 *M* (*SD*) = 54.66 (16.17), T1 *M* (*SD*) = 44.95 (18.14); Control: T0 *M* (*SD*) = 42.96 (19.33), T1 *M* (*SD*) = 44.03 (16.87); between-group *d* = 0.05 [-0.66, 0.77]; partial *η²* = 0.87). †† ‡‡ | Attendence: 100%. Assessed for eligibility: *N* = not stated. Inclusion criteria met: *N* = not stated. EG: T0: *N* = 15; T1: *N* = 15; T2: *N* = 15. CG: T0: *N* = 15; T1: *N* = 15; T2: *N* = 15. |  |

*Notes.* The studies are arranged following the three depicted subpopulations. Abbreviations: AAQ-II, Acceptance and Action Questionnaire-II; CFS, Cognitive Flexibility Scale; CG, Control Group; CompACT, Comprehensive Assessment of Acceptance and Commitment Therapy Processes; DASS, Depression Anxiety Stress Scales; EG, Experimental Group; HB, Health Behavior Engagement Questionnaire; HRQL, PedsQL Parent health-related quality of life; GAD-7, Generalized Anxiety Disorder Scale-7; NSSR, Neuropsychological Symptom Self-Report; NT, No Treatment; PAT, Psychosocial Assessment Tool; PECI, Parent Experience of Child Illness; PHQ-9/4, Patient Health Questionnaire-9/4; PCL-5/C Posttraumatic Stress Disorder (PTSD)-5 / Checklist/Compact; QoL, Quality of life; RNTQ-3, Repetitive Negative Thoughts Questionnaire-3; SDQ, Strengths and Difficulties Questionnaire; TAU, Treatment as Usual; VQ-3, Valuing Questionnaire-3; WL, Waitlist. † Deviation from standard depiction, since means and *SD*s at T0, T1 and T2 were not reported. ‡ In the demographic table is stated *N* = 9 but then describes the age of 10 participants. In the study is stated *N* = 11 at T0 and *N* = 9 at T1. § Calculated based on T2 sample sizes. ¶ Allocated: *N* = 147, completed T0 survey: *N* = 142. † † Control group data at T2 not provided, between group d at T2 not measurable. ‡‡ On the DTS, higher scores indicate greater distress tolerance. The reported descriptive statistics therefore indicate a decrease in distress tolerance in the intervention group.
